# Integrin CD11b activation drives anti-tumor innate immunity

**DOI:** 10.1038/s41467-018-07387-4

**Published:** 2018-12-19

**Authors:** Michael C. Schmid, Samia Q. Khan, Megan M. Kaneda, Paulina Pathria, Ryan Shepard, Tiani L. Louis, Sudarshan Anand, Gyunghwi Woo, Chris Leem, M. Hafeez Faridi, Terese Geraghty, Anugraha Rajagopalan, Seema Gupta, Mansoor Ahmed, Roberto I. Vazquez-Padron, David A. Cheresh, Vineet Gupta, Judith A. Varner

**Affiliations:** 1 Moores Cancer Center, University of California, San Diego, La Jolla, CA 92093 USA; 2 Drug Discovery Center, Department of Internal Medicine, Rush University Medical Center, Chicago, IL 60612 USA; 3 Department of Pathology, University of California, San Diego, La Jolla, CA 92093 USA; 4 Department of Radiation Oncology, University of Miami Miller School of Medicine, Miami, FL 33136 USA; 5 Department of Surgery, University of Miami Leonard M. Miller School of Medicine, Miami, 33136 USA

## Abstract

Myeloid cells are recruited to damaged tissues where they can resolve infections and tumor growth or stimulate wound healing and tumor progression. Recruitment of these cells is regulated by integrins, a family of adhesion receptors that includes integrin CD11b. Here we report that, unexpectedly, integrin CD11b does not regulate myeloid cell recruitment to tumors but instead controls myeloid cell polarization and tumor growth. CD11b activation promotes pro-inflammatory macrophage polarization by stimulating expression of microRNA *Let7a*. In contrast, inhibition of CD11b prevents *Let7a* expression and induces cMyc expression, leading to immune suppressive macrophage polarization, vascular maturation, and accelerated tumor growth. Pharmacological activation of CD11b with a small molecule agonist, Leukadherin 1 (LA1), promotes pro-inflammatory macrophage polarization and suppresses tumor growth in animal models of murine and human cancer. These studies identify CD11b as negative regulator of immune suppression and a target for cancer immune therapy.

## Abstract (web summary)

Recruitment of myeloid cells can be regulated by integrin CD11b. Here the authors show that in the tumor microenvironment, CD11b is not essential for recruitment of myeloid cells but rather induces macrophage anti-tumorigenic polarization via stimulating *let7a* and NFκB signaling and that pharmacological activation of CD11b enhances survival in mouse models of cancer.

## Introduction

Macrophages, monocytes, neutrophils, and other myeloid cells play important roles during acute and chronic inflammation. Pro-inflammatory myeloid cells stimulate cytotoxic T cells to suppress infectious disease and tumor growth, while immune suppressive myeloid cells promote tumor progression and wound healing^1–4^. During acute and chronic inflammation, macrophages express pro-inflammatory cytokines, as well as reactive nitrogen and oxygen species, that can kill pathogens as well as normal cells^3^. In contrast, in neoplastic and parasitic diseases, macrophages and immature monocytes and granulocytes (myeloid-derived suppressor cells) express cytokines that induce immune suppression, angiogenesis, and cancer progression^5,6^. Macrophages isolated from murine and human tumors exhibit a primarily immunosuppressive phenotype^7^.

Although it is well established that tumor-associated macrophages (TAM) are abundant within the tumor microenvironment and play essential roles in tumor immune suppression and progression^1–7^, the molecular mechanisms that regulate these tumor-promoting functions of TAMs remain incompletely clear. However, recent studies have shown that signaling pathways regulated by integrins, CSF1R, PI3Kγ, and BTK control myeloid cell trafficking into tumors as well as macrophage polarization and inhibitors of these molecules are in clinical development for cancer therapy^8–15^.

Myeloid cells as well as lymphocytes rely on cell adhesion receptors for trafficking into inflamed tissues and tumors^2,13–15^. Our previous studies revealed that immune cell adhesion receptors play critical roles during tumor progression. We found that the integrin α4β1, a receptor for fibronectin and VCAM-1, is required for myeloid cell trafficking into tumors as well as subsequent tumor progression^13–15^. These same studies found that the myeloid cell integrin, αMβ2 (CD11b/CD18), a receptor for complement, fibrinogen, and endothelial cell ICAM-1, is not required for adhesion to endothelium or trafficking into tumors^15^. In contrast, CD11b/CD18 has been shown to mediate macrophage adhesion, migration, chemotaxis and accumulation during inflammation^16–18^. As integrin CD11b plays important roles during inflammation, we set out to identify whether this integrin regulates immune responses during tumor progression.

We report here that the integrin CD11b/CD18 regulates macrophage polarization by promoting miR-*Let7a*-dependent pro-inflammatory macrophage transcription, thereby restraining immunosuppressive macrophage polarization. Using genetic and pharmaceutical approaches, we show that CD11b signaling inhibits immune suppression, modulates neovascularization and promotes anti-tumor immune responses in models of murine and human cancer. We also show that a small molecule CD11b agonist, Leukadherin 1, inhibits anti-inflammatory macrophage polarization to suppress tumor growth and enhance survival in animal models of murine and human cancer.

## Results

### Integrin CD11b regulates macrophage polarization

**Fig. 1:**
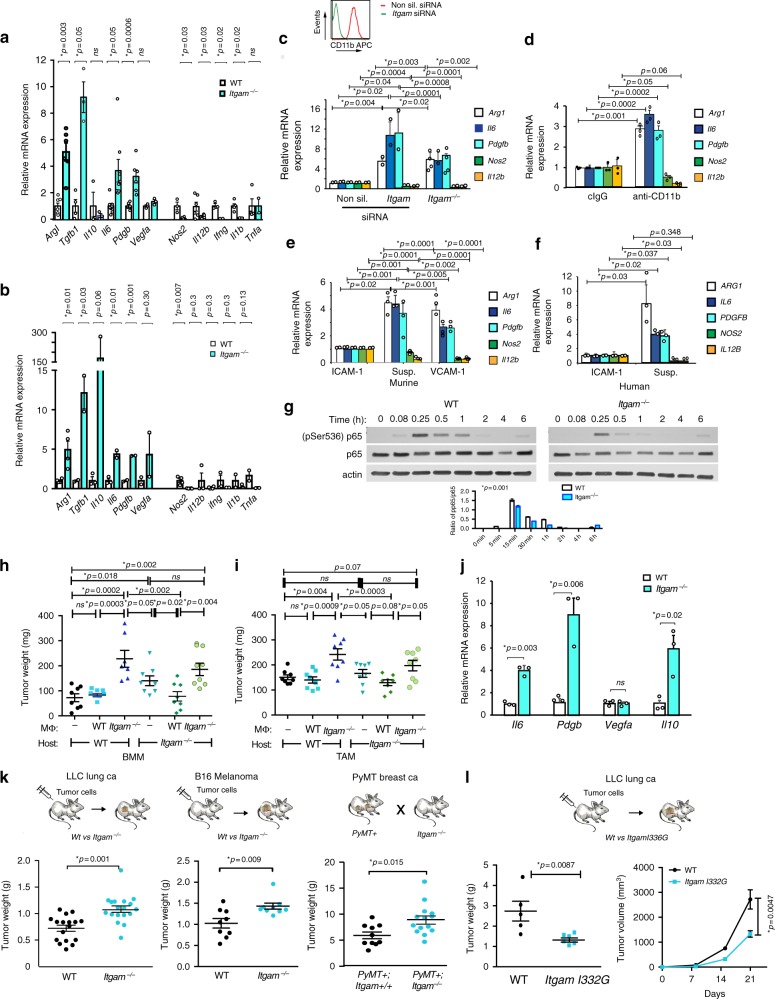
CD11b ligation promotes pro-inflammatory macrophage signaling. **a**–**f** Relative mRNA expression of pro- and anti-inflammatory cytokines in **a** bone marrow derived macrophages (BMDM) from WT (white bars) or *Itgam*−/− (cyan bars) mice (*n* = 2–8); **b** tumor associated macrophages (TAM) from WT (white bars) and *Itgam−/−* (cyan bars) mice bearing LLC lung carcinoma tumors (*n* = 2–4); **c** *Itgam−/−* and *Itgam* or non-silencing siRNA transfected macrophages (*n* = 2–4); inset: cell surface expression levels of CD11b in transfected macrophages; **d** WT macrophages in the presence of non-specific (IgG) or anti-CD11b antibodies (*n* = 3), **e** murine macrophages adherent to ICAM-1, VCAM-1 or BSA coated plates (Susp) (*n* = 3) and **f** human macrophages adherent to ICAM-1 or BSA coated plates (Susp) (*n* = 3). **g** Immunoblotting of phosphoSer536 and total p65 NFκB RelA in WT and *Itgam−/−* macrophages stimulated with IFNγ + LPS; graph depicts quantification of relative pSer536 expression in WT (white bars) and *Itgam*−/− (blue bars) BMDM. **h**, **i** LLC tumor growth in WT and *Itgam*-/- mice adoptively transferred with **h** bone marrow derived and **i** tumor derived WT or *Itgam−/−* macrophages. **j** Relative mRNA expression of cytokines in whole LLC tumors from WT (white bars) and *Itgam*−/− (cyan bars) mice (*n* = 3). **k** LLC lung (*n* = 17), B16 melanoma (*n* = 9) and autochthonous PyMT mammary (*n* = 10–14) tumor weight in WT (black dots) and *Itgam−/−* (cyan dots) mice. **l** Tumor weight and volume of LLC tumors grown in WT (black dots) versus *Itgam* I332G knockin mice (cyan dots) (*n* = 6–7). Error bars indicate sem. “*n*” indicates biological replicates. *p* < 0.05 indicates statistical significance determined by Student’s *t*-test for **a**–**g** and **j**. and by Anova with Tukey post-hoc testing for **h**, **i**, **k**, **l**. Source data are provided in Source Data file

We previously reported that the VCAM receptor integrin α4β1 promotes myeloid cell recruitment from the bone marrow to the tumor microenvironment, thereby stimulating immune suppression, angiogenesis and tumor progression^2,13–15^. In contrast to its role in regulating recruitment of myeloid cells to tissues during acute inflammation^16–18^, we found that CD11b (αMβ2), a myeloid cell integrin receptor for ICAM-1 and fibrinogen, does not affect myeloid cell recruitment to tumors, as global deletion of CD11b in *Itgam−/*− mice has no effect on the number of myeloid cells in circulation or in the number of cell recruited to tumors (Supplementary Figure 1; Supplementary Figure 2a-d). Surprisingly, however, we found that integrin CD11b plays an essential role in regulating macrophage polarization. *Itgam*−/− macrophages exhibited enhanced immune suppressive gene and protein expression and strongly reduced pro-inflammatory gene and protein expression compared with WT macrophages, whether stimulated under basal, IL-4 or IFNγ/LPS stimulation conditions (Fig. 1a, Supplementary Figure 2e-f). To determine whether CD11b also regulates macrophage polarization in vivo, we isolated and characterized F4/80 + TAMs from LLC tumors grown in *Itgam*−/− and WT mice. We found that *Itgam*−/− TAMs also expressed significantly higher levels of mRNAs associated with immune suppression and angiogenesis, such as *Arg1*, *Tgfb*, *Il10*, *Il6,* and *Pdgfb*, and significantly lower expression of genes associated with immune stimulation, such as *Ifng*, *Nos2*, and *Tnfa* than did WT TAMs (Fig. 1b, Supplementary Figure 2g).

Importantly, transient siRNA-mediated knockdown of CD11b in in vitro cultured macrophages elevated immune suppressive gene expression and decreased immune stimulatory gene expression, effects that are comparable to CD11b deletion (Fig. 1c), indicating that even transient loss of CD11b controls macrophage immune suppressive gene expression. To address whether CD11b expression or function controls macrophage gene expression, we examined the effect of inhibitory CD11b antibodies on macrophage mRNA expression. Blockade of murine macrophage CD11b mediated attachment to ICAM-1-coated substrates by anti-CD11b neutralizing antibodies also induced immune suppressive mRNA expression in macrophages (Fig. 1d). Similarly, adhesion of macrophages to the integrin α4β1 substrate VCAM-1 or loss of attachment by suspension culture promoted murine and human immune suppressive transcription, while attachment to ICAM-1 coated surfaces promoted immune stimulatory transcription (Fig. 1e, f, Supplementary Figure 1h), indicating that ligation of CD11b controls immune stimulatory macrophage transcription.

The loss of pro-inflammatory cytokine expression in *Itgam−/*− macrophages suggested that CD11b may regulate activation of pro-inflammatory transcription factors, such as NFκB. We found that *Itgam*−/− macrophages exhibited reduced NFκB serine 536 phosphorylation (an indication of reduced activation^19^) in response to LPS stimulation compared to WT macrophages, suggesting CD11b plays a role in NFκB activation (Fig. 1g). As other studies have implicated CD11b in the promotion of pro-inflammatory responses of monocytes and dendritic cells through direct interactions of LPS with integrin beta2 extracellular domains^20,21^, our results indicate that CD11b activation and signaling play key roles in the regulation of macrophage polarization in vitro and in vivo.

### Macrophage CD11b regulates tumor growth

Our data indicate that *Itgam−/−* bone marrow derived and tumor associated macrophages exhibit more immune suppressive transcriptional profiles than WT macrophages. To determine if this difference affects tumor growth, we adoptively transferred WT or *Itgam−/−* bone marrow derived or tumor associated macrophages with tumor cells into recipient WT or *Itgam−/*− mice. Previously, we demonstrated that adoptively transferred, immune suppressive BMDM or TAMs can stimulate tumor growth^9,10^. Remarkably, bone marrow-derived *Itgam−/−* macrophages (Fig. 1h) as well as tumor-derived *Itgam*−/− macrophages (Fig. 1i) potently stimulated tumor growth compared with WT macrophages in both WT and *Itgam−/−* mice. As *Itgam−/−* macrophages exhibit an immune suppressive transcriptional profile (Fig. 1b), and tumors derived from *Itgam−/*− mice exhibit an overall immune suppressive transcriptional profile (Fig. 1j), these data suggested that CD11b expression or activation might impact overall tumor growth. Indeed, we found that subcutaneous (LLC), orthotopic (melanoma) and autochthonous (PyMT) tumors grew more aggressively in *Itgam*−/− than in WT mice (Fig. 1k). As *Itgam*−/− mice exhibited substantially more CD4 + Foxp3 + T_regs_ and fewer CD8+ T cells in tumors than WT mice (Supplementary Figure 3a-b), our studies support the conclusion that CD11b plays a key role in regulating the overall immune response in tumors.

Prior studies have shown that Isoleucine 332 in the CD11b molecule serves as an allosteric switch controlling the adhesion receptor’s activation and shape^22^. To determine whether CD11b activation controls tumor development, we generated a constitutively activated CD11b knockin mouse strain (C57BL/6 ITGAM^*I332G*^ by introducing an I332G point mutation in the murine *Itgam* gene. I332G knockin mice express normal levels of cell surface CD11b on both monocytes and granulocytes and exhibit normal levels of all blood cell level (Supplementary Figure 3c-d). In vitro adhesion assays with bone marrow derived macrophages from these mice showed that I332G cells express constitutively active CD11b (Supplementary Figure 3e). Importantly, I332G *Itgam* knockin mice exhibited significantly reduced LLC tumor growth (Fig. 1k). Thus, while CD11b deletion stimulates anti-inflammatory macrophage polarization, inhibits CD8+ T cell recruitment and promotes tumor growth, CD11b activation potently inhibits tumor growth. These studies indicate that macrophage CD11b plays a critical functional role in controlling tumor growth.

### Immune suppressive signals inhibit CD11b expression

To determine whether signals associated with the tumor microenvironment can alter CD11b expression and subsequently affect myeloid cell polarization, we evaluated the effect of macrophage media (mCSF-, IL-4- and IFNγ/LPS) on cell surface CD11b expression in bone marrow derived macrophages. While the immune suppressive cytokine IL-4 reduced CD11b expression, the pro-inflammatory stimuli IFNγ/LPS enhanced CD11b surface expression compared to levels expressed on mCSF-stimulated macrophages (Supplementary Figure 4a-b). Additionally, the immune suppressive factor TGFβ, but not IL-10, inhibited CD11b surface expression (Supplementary Figure 4c); TGFβ, IL-4 and tumor cell conditioned medium (TCM) each also suppressed *Cd11b* mRNA expression (Supplementary Figure 4d). Importantly, TGFβ and TCM reduced CD11b cell surface expression and stimulated immune suppressive transcription while inhibiting immune stimulatory transcription in a manner that was reversed by the TGFβR1 inhibitor SB525334 (Supplementary Figure 4e-g). These data indicate that cytokines such as TGFβ in the tumor microenvironment suppress CD11b expression or activation, thereby promoting immune suppressive macrophage polarization.

### Macrophage CD11b regulates blood vessel stability

**Fig. 2:**
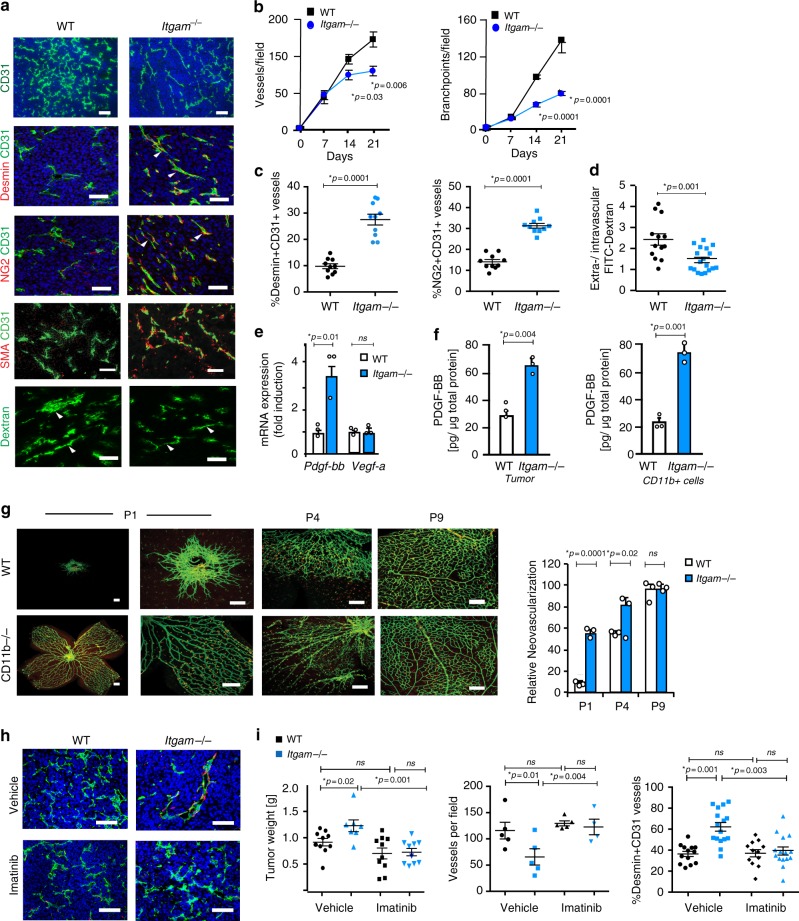
CD11b suppresses PDGF-BB-dependent neovascularization and tumor growth. **a** CD31, Desmin, NG2, Smooth muscle actin (SMA) and Dextran immunostaining of blood vessels in LLC tumors from WT (white bars) and *Itgam*−/− (blue bars) mice (*n* = 10). **b** Blood vessel density (vessels/field) and number of vessel branch points/field in LLC tumors from WT (white bars) and *Itgam*−/− (blue bars) animals (*n* = 10). **c** Percent CD31+/SMA+, CD31+/Desmin+, CD31+/NG2+ vessels in tumors from WT (white bars) and *Itgam*−/− (blue bars) (*n* = 5). **d** Ratio of extravascular to intravascular FITC-dextran in LLC tumors from WT (white bars) and *Itgam*−/− (blue bars) mice (*n* = 5). **e**, **f** *Pdgfb* and *Vegfa* **e** mRNA (*n* = 3) and **f** PDGF-BB protein expression (*n* = 12) in LLC tumors from WT (white bars) and *Itgam*−/− (blue bars) mice (*n* = 3). **g** Left, FITC-Isolectin staining of whole mount retinas from newborn WT (white bars) and *Itgam*−/− (blue bars) mice. Right, Percent neovascularization at P1, P4, and P9 retinas in WT (white bars) and *Itgam*-/- (blue bars) mice (*n* = 5). **h** Desmin (red) and CD31 (green) immunostaining of LLC tumors from Imatinib and vehicle-treated WT (white bars) and *Itgam*-/- (blue bars) mice. **i** Tumor weight, number of blood vessels/field and percent Desmin/CD31 vessels per field from **h** (*n* = 10). Bar on micrographs indicates 50 µm. Error bars indicate sem. “*n*” indicates biological replicates. *p* < 0.05 indicates statistical significance, as determined by Student’s *t*-test for **b**–**g** and by Anova with Tukey’s post-hoc testing for **i**. Source data are provided in Source Data file

Macrophages not only control immune responses but also angiogenesis and desmoplasia, by expressing cytokines such as VEGF-A and PDGF-BB, growth factors that regulate endothelial cell and vascular smooth muscles/pericytes during angiogenesis, respectively^2^. Tumor blood vessels often consist of a single endothelial layer that lacks supporting pericytes or smooth muscle cells; these blood vessels are more numerous in tumors than in normal tissues but are aberrantly formed and poorly perfuse. In contrast, in tumors with high PDGF to VEGF ratios, blood vessels are lined by pericytes, mesenchymal cells that stabilize vessels and promote better tumor perfusion^23–27^. These tumors grow more rapidly than tumors with lower PDGF to VEGF ratios but also respond better to chemotherapy and immune therapy due to better tumor perfusion^24–36^. As *Itgam−/−* macrophages exhibited high PDGF and low VEGF gene expression (Fig. 1a, b), we examined the patterns of blood vessel development in *Itgam−/*− and WT tumors. An assessment of vascular patterning in LLC and PyMT tumors from WT and *Itgam*−/− mice showed that *Itgam*−/− tumors exhibited fewer, longer blood vessels with wider lumens and fewer branch points/field than WT tumors (Fig. 2a, b; Supplementary Figure 5a). *Itgam*−/− tumors had more blood vessels that were lined with Desmin+, NG2+, or SMA+ pericytes/smooth muscle cells than did WT tumors (Fig. 2a–c; Supplementary Figure 5b). Accordingly, these vessels were less permeable in *Itgam*-/- mice than in WT mice, as less intravascular FITC-dextran leaked into the tumor parenchyma (Fig. 2a–d). These data indicate that the integrin CD11b plays a role in controlling blood vessel maturation. We found that gene expression of PDGF-BB but not VEGF-A was strongly enhanced in tumors (Fig. 2e; Supplementary Figure 5c). Importantly, PDGF-BB protein expression was also elevated in *Itgam*−/− tumors and tumor-derived macrophages compared to WT tumors (Fig. 2f). Together, these data suggest that macrophage CD11b controls tumor vascularization through the constitutive expression of elevated levels of PDGF.

In support of these observations on CD11b roles in neovascularization, we found that *Itgam*−/− mice exhibited a well-developed retinal vascular plexus (Fig. 2g, Isolectin B+, green) at birth (P1) compared with WT mice, which exhibit undeveloped retinal vasculature that expands progressively from postnatal day 1 (P1) to P9. The superficial vascular plexus was more developed in *Itgam*−/− mice from postnatal day P1 through postnatal day P9 than in WT neonates (Fig. 2g). These results indicate that macrophages and CD11b play key roles in the control normal vascular patterning.

To investigate whether elevated PDGF-BB is responsible for the enhanced vascular maturation and tumor growth in *Itgam*−/− mice, we treated WT and *Itgam*−/− mice bearing LLC tumors with imatinib, an inhibitor of the PDGF-BB receptor PDGFR1. Imatinib treatment suppressed the enhanced tumor growth observed in *Itgam−/*− mice (Fig. 2h, i). It also increased vascular density and suppressed vascular normalization in *Itgam−/−* mice (Fig. 2h, i). Together these results support the concept that integrin CD11b modulates vascular development through control of PDGF-BB expression.

### CD11b regulates Let7a and c-Myc expression

**Fig. 3:**
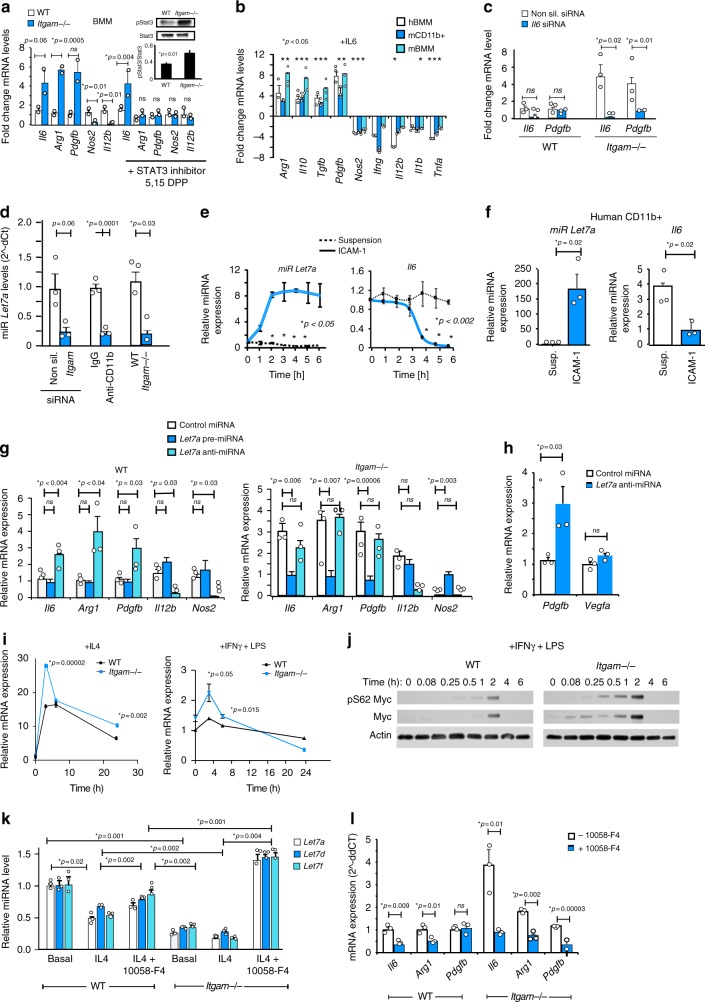
CD11b promotes miR-*Let7a* mediated immune stimulation. **a** Relative mRNA expression of pro-inflammatory and anti-inflammatory factors in WT (white bars) and *Itgam*−/− (blue bars) macrophages incubated with and without the Stat3 inhibitor 5,15 DPP; inset, Stat3 phosphorylation in WT and *Itgam*−/− macrophages (*n* = 2–3). **b** Relative mRNA expression of pro-inflammatory and anti-inflammatory factors in IL6-stimulated human (white bars) and murine (cyan bars) bone marrow-derived macrophages and murine total bone marrow derived myeloid cells (blue bars) (*n* = 3); *p* < 0.05 with these exceptions: mBMM (*Ifng*, Il12b); mCD11b+ (*Arg1*, *Pdgfb*, *Il12b* and *Il1b);* hBMM (*Arg1, Ifng, Il1b*). **c** Relative *Il6* and *Pdgfb* mRNA expression in WT and *Itgam*−/− cells transduced with non-silencing (white bars or *Il6* (blue bars) siRNA (*n* = 3). **d** Relative *Let7a* expression in murine macrophages transduced with non-silencing (white bars) or *Itgam* (blue bars) siRNAs, macrophages incubated with control IgG (white bars) or neutralizing anti-CD11b (blue bars) antibodies, and WT (white bars) or *Itgam*−/− (blue bars) macrophages (*n* = 3). **e** Time course of *Let7a* (left) and *Il6* (right) expression in WT murine CD11b+ cells seeded on ICAM-1 (blue solid line) or maintained in suspension (black dotted line) (*n* = 3). **f** Relative expression of miRNA *Let7a* and *Il6* in human macrophages adherent to ICAM-1 (blue) or maintained in suspension (white) (*n* = 3). **g** Relative mRNA expression of inflammatory factors in WT and *Itgam*-/- BMM transduced with control (white bars), pre-miRNA *Let7a* (cyan bars) or anti-miRNA *Let7a* (blue bars) (*n* = 3). **h** Relative *Pdgfb* and *Vegfa* expression in WT BMM transduced with control (white bars) or anti-miRNA *Let7a* (blue bars) (*n* = 3). **i** Time course of *c-Myc* expression in IL-4 or IFNγ + LPS stimulated WT (black lines) or *Itgam*−/− (blue lines) macrophages (*n* = 3). **j** Time course of c-Myc expression and pSer62myc phosphorylation in WT and *Itgam*−/− macrophages. **k** Relative mRNA expression of miRNAs *Let7a* (white bars), *Let7d* (blue bars) and *Let7f* (cyan bars) in basal, IL-4, or IL-4+ c-myc inhibitor treated WT and *Itgam*−/− macrophages (*n* = 3). **l** Relative mRNA expression of *Il6*, *Arg1*, and *Pdgfb* in IL-4 stimulated WT (white bars) and *Itgam*−/− (blue bars) macrophages treated with (blue bars) or without (white bars) c-Myc inhibitor 10058-F4. Error bars indicate sem. “*n*” indicates biological replicates. *p < 0.05 indicates statistical significance determined by Student’s *t*-test for **a**, **d**–**f**, and Anova with Tukey’s post-hoc testing for **b**, **g**, **k**. Source data are provided as a Source Data file

CD11b may control anti-inflammatory macrophage polarization through the activation of transcription factors such as Stat3, which can promote expression of immune suppressive and pro-angiogenic factors such as Arginase 1, Myc, and VEGF^37–39^. *Itgam−/*− macrophages exhibit constitutively phosphorylated Stat3 (Fig. 3a inset); the high levels of immune suppressive factor expression in *Itgam−/−* macrophages were reduced to WT macrophage levels by treatment with the Stat3 inhibitor 5,15-DPP (Fig. 3a). Surprisingly, however, Stat3 inhibition did not affect the high levels of *Il6* expression observed in *Itgam*−/− macrophages (Fig. 3a). Importantly, IL-6 can directly activate Stat3^38^. We found that IL-6 promoted the same pattern of immune suppressive polarization in murine and human myeloid cells and macrophages we observed in *Itgam-/-* macrophages (Fig. 3b). Together, these results suggested that autocrine IL6 may drives the constitutively immune suppressive polarization observed in *Itgam−/*− macrophages. In support of this concept, *Il6* knockdown decreased expression of constitutive *Pdgfb* expression in *Itgam*−/− macrophages (Fig. 3c). As TAMs are a major source of *Il6* expression in tumors^15^, these results suggest that CD11b serves as a natural brake on immune suppression in part through control of myeloid cell transcription of *Il6*.

The *Let7* family of microRNAs can controls *Il6* expression in tumor and inflammatory cells^40,41^. microRNAs are non-coding RNAs that modulate gene expression at the post-transcriptional level by interfering with RNA translation or stability and can dramatically impact tumor immune suppression and angiogenesis^42,43^. We found that miRNA *Let7a* expression inversely correlated with *Il6* expression in murine and human macrophages (Supplementary Figure 6a-b). Therefore, we asked whether loss of CD11b expression in macrophages affects Let7a expression. *Let7a* expression was ablated in *Itgam−/−* and *Itgam* siRNA transduced macrophages and in in the presence of neutralizing CD11b antibodies (Fig. 3d; Supplementary Figure 6c) in a manner that was independent of Lin28, an RNA binding protein that cleaves and inactivates Let7, as Lin28 levels were not affected by CD11b expression or activation (Supplementary Figure 6b, d-e). CD11b ligation by ICAM-1 promoted time-dependent *Let7a* expression, while inhibiting *Il6* expression; conversely, suppression of adhesion inhibited *Let7a* expression and promoted *Il6* expression in both murine and human macrophages (Fig. 3e, f). Importantly, ectopic expression of *Let7a* miRNA (pre-miRNA) inhibited immune suppressive gene expression and stimulated pro-inflammatory gene expression in *Itgam*−/− macrophages, while anti-miRNA *Let7a* stimulated immune suppressive gene expression and inhibited immune stimulatory gene expression in WT macrophages (Fig. 3g). Similar to CD11b ablation (Fig. 1a), anti-miRNA *Let7a* stimulated *Pdgfb* expression but had no effect on *Vegfa* expression (Fig. 3h). Together, these results indicate that CD11b activation promotes miRNA *Let7a* expression, which in turn inhibits IL6-mediated immune suppressive macrophage gene expression.

c-Myc, a transcription factor that regulates immune suppressive macrophage polarization, binds to the *Let7* promoter and suppresses its transcription; interestingly *Let7* can also suppress *c-Myc* expression^44,45^. We found that *c-Myc* gene was upregulated in *Itgam*−/− macrophages compared with WT macrophages (Fig. 3i). c-Myc protein expression and serine 62 phosphorylation, which stabilizes the transcription factor^46^, were also upregulated in *Itgam*−/− macrophages compared with WT macrophages (Fig. 3j). We then asked whether inhibition of cMyc function could promote Let7 expression and thereby alter macrophage polarization. Importantly, *Let7a*, *Let7d*, and *Let7f* expression was reduced in *Itgam−/−* macrophages; however, pharmacological inhibition of c-Myc restored *Let7* expression in *Itgam−/*− macrophages and reversed the increased immune suppressive gene expression exhibited by *Itgam−/−* macrophages (Fig. 3k, l). Together, these data indicate that integrin CD11b functions to suppress Myc expression and immune suppressive macrophage polarization in a *Let7* dependent manner.

**Fig. 4:**
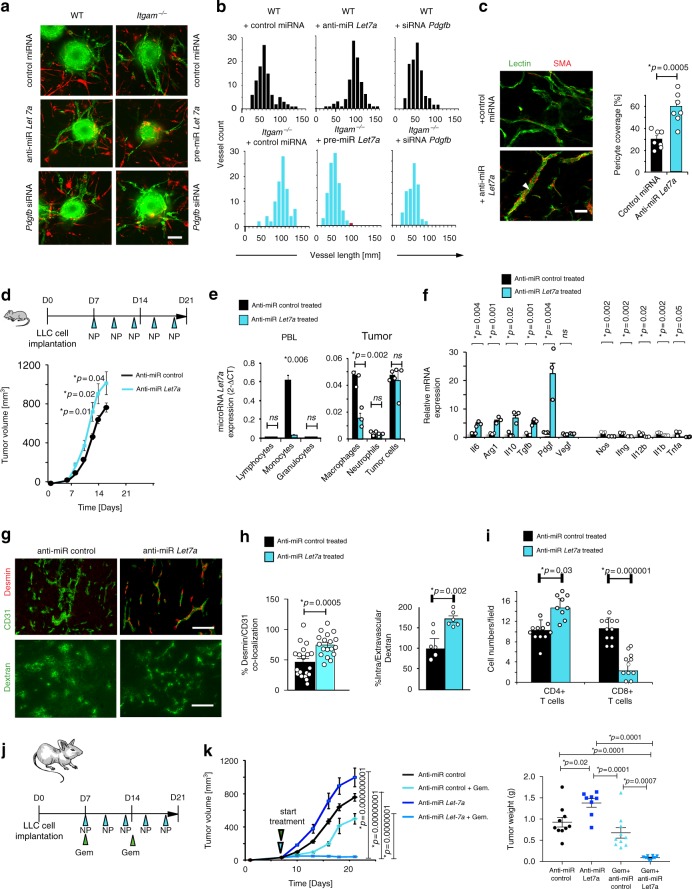
Macrophage microRNA *let-7a* is required for tumor growth suppression. **a**, **b** Endothelial cells and vascular smooth muscle cells attached to microcarrier beads were cultured in fibrin gels containing WT or *Itgam*−/− BMMs transduced with control miRNA, pre-miRNA *Let7a*, anti-miRNA *Let7a* or *Pdgf-bb* siRNA. **a** Images **b** histograms of CD31+ positive vessel length (mm) (*n* = 10). **c** CD31 (green) and SMA (red) immunostaining of sections from in vivo cultured bFGF-saturated Matrigel plugs containing BMM transduced with control miR (black bars) or anti-miR *Let7a* (blue bars); quantification of the percentage of SMA+ vessels per matrigel plug (*n* = 25). **d** Schematic and graph of targeted delivery of anti-miR *Let7a* in animals with LLC tumors; tumor volumes from control anti-miRNA (black line) or anti-miRNA *Let-7a* (cyan line) treated animals (*n* = 10). **e** *Let7*a expression in cell populations sorted from peripheral blood cell and tumors from control (black bars) and anti-miRNA Let7-treated (cyan bars) animals from **d** (*n* = 3). **f** Relative mRNA expression of inflammatory factors in sorted macrophages from **d** (*n* = 3). **g** Representative images of CD31/Desmin co-localization and FITC-Dextran localization in treated tumors from **d**. **h** Quantification of the percentage of CD31/Desmin co-localization (*n* = 30) and of FITC-dextran leakage into tissues (*n* = 25). **i** CD4+ and CD8+ cells/field in tumors from control anti-miR (black bars) or anti-miR *let-7a* (blue bars) transduced animals (*n* = 25) scale bars, 40 µm. **j** Schematic representation of chemotherapeutic treatment in combination with targeted delivery of anti-miR-let7a. **k** Volumes and endpoint weights of LLC tumors in animals transduced with control anti-miR (black), anti-miR let-7a (blue), control anti-miR /Gemcitabine (green), and anti-miR let7a/Gemcitabine (red) (*n* = 10). Bar on micrographs indicates 50 µm. Error bars indicate sem. “*n*” indicates biological replicates. *p < 0.05 indicates statistical significance by Student’s *t*-test for **c**–**i** and by Anova with Tukey’s post-hoc testing for **j** Source data are provided as a Source Data file

Because *Let7a* inhibits macrophage-mediated *Pdgfb* expression, we investigated the effect of *Let7a* expression on neovascularization in vitro and in vivo. Endothelial cells and vascular smooth muscle cells attached to microcarrier beads were cultured in fibrin gels that contained either WT or *Itgam−/−* macrophages that were transduced with control miRNA, pre-miRNA *Let7a*, anti-miRNA *Let7a* or *Pdgfb-bb* siRNA. *Itgam*−/− macrophages stimulated sprout elongation that was inhibited by transduction of macrophages with *Let7a* miRNA or *Pdgfb* siRNA (Fig. 4a, b; Supplementary Figure 6f). In contrast, expression of anti-miRNA *Let7a* in WT but not *Itgam*-/- macrophages stimulated sprout elongation (Fig. 4a, b; Supplementary Figure 6f). Additionally, macrophages transduced with anti-miR *Let7a* stimulated the formation of mature, pericyte-coated blood vessels in bFGF-saturated Matrigel in vivo (Fig. 4c). Together, these studies show that CD11b controls neovascularization through the regulation of *Let7a* and subsequent PDGF-BB expression.

### Myeloid cell Let7a regulates tumor progression

To test the role of *Let7a* in the regulation of tumor immune suppression and neovascularization, we delivered anti-miR *Let7a* to tumors in myeloid cell targeted nanoparticles in vivo (Fig. 4d). We found that integrin αvβ3-targeted nanoparticles were specifically taken up by circulating myeloid cells in normal and tumor bearing animals (Supplementary Figure 7a-h). Delivery of anti-miRNA *Let7a* stimulated LLC tumor growth, comparable to that observed in *Itgam−/−* mice (Fig. 4d). Although *Let7a* is expressed in immune and non-immune cells in tumors, we found that delivery of anti-miR *Let7a* only inhibited *Let7a* expression in circulating monocytes and in tumor associated macrophages but not in other tumor associated cells (Fig. 4e). Importantly, anti-miRNA *Let7a* stimulated immune suppressive and pro-angiogenic gene expression and inhibited pro-inflammatory gene expression in tumors compared with controls (Fig. 4f, Supplementary Figure 8a). Anti-miRNA *Let7a* also stimulated blood vessel normalization in transfected tumors, as vessels were longer, less branched, heavily coated with pericytes and less leaky than vessel from control transfected tumors (Fig. 4g, h). Importantly, anti-*Let7a* also suppressed CD8+ T cell recruitment to tumors and enhanced CD4+ T cell recruitment to tumors (Fig. 4i). Together, these results indicate that CD11b restrains immune suppression and vascular maturation through its regulation of miRNA *Let7a*. Prior studies have shown that increased vascular normalization in tumors can improve tumor perfusion and promote responsiveness to therapy^23–36^. To determine whether the vascular normalization induced by anti-miRNA *Let7a* might enhance the efficacy of chemotherapy by increasing tumor perfusion, we treated mice bearing LLC tumors with targeted delivery of anti-miRNA *Let7a* or control miRNA in combination with chemotherapy (gemcitabine) (Fig. 4j). Whereas anti-miRNA *Let7a* promoted LLC tumor growth, anti-miRNA *Let7a* combined with gemcitabine substantially reduced tumor growth, consistent with the notion that *Let7a* inhibition increases accessibility of the tumor to chemotherapy (Fig. 4k). In accordance with these results, we found that gemcitabine treatment of *Itgam−/−* mice suppressed tumor growth more profoundly than gemcitabine treatment of WT mice (Supplementary Figure 8b). As *Itgam−/−* exhibited greater perfusion (less vascular leak) than WT mice (Supplementary Figure 8c), these studies indicate that CD11b, through its effects on miRNA Let7a, plays a critical role in regulating tumor immune and vascular responses.

### The CD11b agonist LA1 inhibits tumor growth

**Fig. 5:**
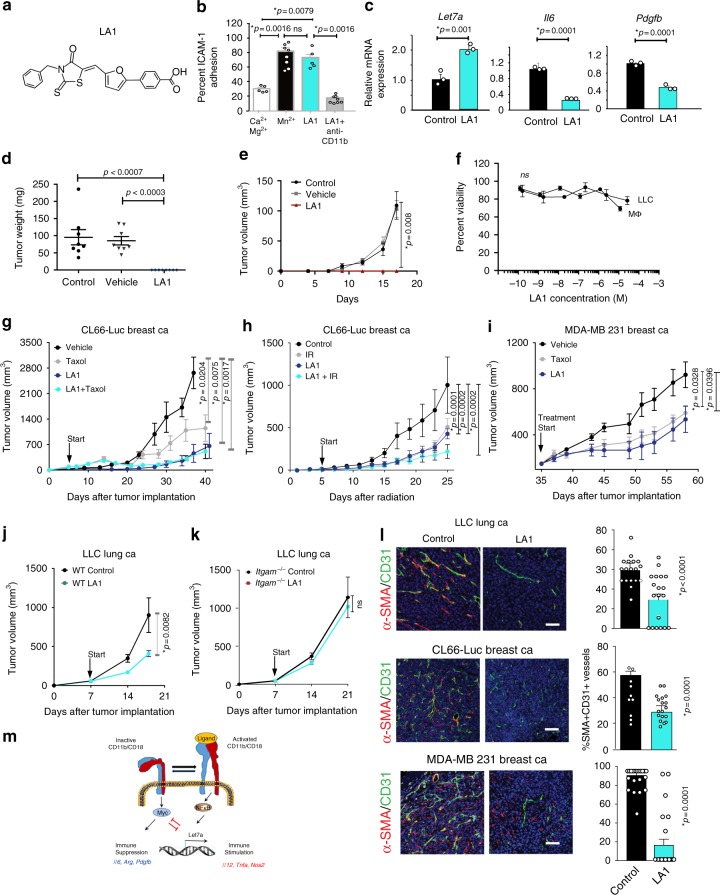
Integrin CD11b agonism suppresses tumor growth and promotes survival in mouse models of cancer. **a** Structure of LA1. **b** Adhesion of macrophages in the absence or presence of Ca^2+^Mg^2+^ (white bars) Mn^2+^ (black bars), LA1 (cyan bars) or LA1+ neutralizing anti-CD11b (gray bars) (*n* = 3). **c** Relative mRNA expression of *Let7a*, *Il16*, or *Pdgfb* in control (black bars) and LA1 (cyan bars)-treated macrophages (*n* = 3). **d** Tumor weights 16 days after implantation of LLC cells mixed 1:1 with control-treated (dots), DMSO-treated (triangles) or LA1-treated (diamonds) tumor-derived macrophages (*n* = 8). **e** Tumor growth curves as represented by volumes from **d**: control (black line), vehicle (gray line) and LA1 (red line) (*n* = 8). **f** Effect of LA1 on in vitro proliferation of LLC cells and macrophages (*n* = 4). **g** Tumor volumes of orthotopic CL66 breast tumors treated with vehicle (black line), Taxol (gray line), LA1 (dark blue line) or LA1 + Taxol (cyan line) (*n* = 10–15). **h** Tumor volumes of orthotopic CL66 tumors treated with vehicle (black line), irradiation (gray line) (IR, 20 Gy), LA1 (dark blue line) (2 mg/kg), or LA1 + IR (cyan line) (*n* = 9). **i** Tumor volumes of orthotopic human MDA-MB-231 mammary xenografts treated with vehicle (control, black line), Taxol (gray line), or LA1 (blue line) (*n* = 7). **j,** **k** Mean LLC subcutaneous tumor volumes of **j** WT (black line) and **k** *Itgam-/-* (cyan line) mice treated with and without LA1 (*n* = 6). **l** Images and quantification of SMA/CD31 expression in blood vessels of control (black bars) and LA1 treated (cyan bars) animals from **g**, **i**, and **j**. Bar on micrographs indicates 50 µm. **m** Schematic depicting role of CD11b activation in the control of immune stimulation. Error bars indicate sem. “*n*” indicates biological replicates. *p < 0.5 indicates statistical significance by Student’s *t*-test **c**, **f**; Anova with Tukey post-hoc testing for **d**–**e**; unpaired *t*-test **g**, **i**; Mann–Whitney *t*-test **b**, **j**–**l**; Wilcox test 5 h. Source data are provided as a Source Data file

Our results suggested that targeted pharmacologic activation of CD11b in vivo might repolarize tumor associated macrophages, with subsequent inhibition of tumor immune suppression and tumor growth. We thus investigated the effects of a small molecule agonist of CD11b, leukadherin 1 (LA1)^47,48^ (Fig. 5a) on macrophage polarization and tumor growth. LA1 stimulated myeloid cell adhesion to ICAM-1 coated substrates in a manner that was inhibited by anti-CD11b-neutralizing antibodies (Fig. 5b). LA1 stimulated macrophage immune response gene expression, illustrated by increases in expression of *Il1b, Tnfa, Il12, Nos2*, and *Ifng* mRNAs (Supplementary Figure 9a). As LA1 stimulated *Let7a* expression and inhibited *Pdgfb* and *Il6* expression (Fig. 5c), these results suggested that LA1 might stimulate pro-inflammatory immune responses that could inhibit tumor growth in vivo. To assess the effects of LA1 on tumor associated macrophages in vivo, tumor associated macrophages were isolated^10^, treated with LA1 prior and co-implanted with LLC tumor cells. LA1-treated macrophages completely inhibited tumor growth (Fig. 5d, e) even though LA1 had no direct effect on LLC or macrophage viability (Fig. 5e, f). Although LA1 had no effect on CL66-Luc breast tumor cell growth in vitro (Supplementary Figure 9b), LA1 potently reduced tumor growth in syngeneic, orthotopically implanted CL66-Luc breast tumors more effectively than taxol (Fig. 5g). LA1 also synergized with irradiation to suppress CL66-Luc breast tumor growth (Fig. 5h) and suppressed the growth of orthotopic, human MDA-MB-231 mammary xenograft tumors (Fig. 5i). Importantly, LA1 inhibited murine LLC lung tumor growth in WT but not in *Itgam−/−* mice, indicating that LA1 acts through integrin CD11b to suppress the growth of tumors (Fig. 5j, k).

As LA1 treatment increased the presence of MHC-II + macrophages, typically considered immune competent, and decreased the presence of CD206+ macrophages, typically considered immune suppressive, in LLC and CL66-Luc tumors (Supplementary Figure 9c-d), our studies suggest that LA1 repolarizes tumor associated macrophages. Accordingly, we found that LA1 inhibited expression of S100A8 and MMP9 in CD11b+ cells in LLC tumors and also inhibited expression of Arginase1, S100A8 and MMP9 in CL66-Luc tumors (Supplementary Figure 9e-f). As these proteins are markers of pro-tumoral macrophages, together these studies indicate that LA1 likely inhibits tumor growth by repolarizing tumor associated macrophages. Indeed, LA1 treatment increased the presence of CD8 + T cells in both LLC and CL66-Luc tumors (Supplementary Figure 10a-c). We also observed that LA1 treatment altered neovascularization in tumors by decreasing the numbers of SMA + blood vessels (Fig. 5l). By enhanced the pro-inflammatory immune profile of tumors and inhibiting vascular normalization in tumors, the small molecule CD11b agonist LA1 significantly altered macrophage polarization, increased CD8+ T cell recruitment to tumors and inhibited tumor progression in mouse models of murine and human cancer.

## Discussion

We identified macrophage integrin CD11b as a critical regulator of pro-inflammatory immune responses that prevent cancer progression. Our studies demonstrate that CD11b ligation/activation inhibits the immune suppressive transcriptional signature of tumor-derived macrophages, stimulates accumulation of CD8+ T cells in tumors and suppresses tumor growth. Loss of CD11b expression or function promotes immune suppressive gene expression in macrophages in vitro and TAMs in vivo, increases FoxP3+ CD4+ T cells and decreases CD8+ T cell recruitment to tumors and increases tumor growth. In contrast, activation of CD11b with the small molecule agonist LA1 stimulates macrophage pro-inflammatory transcription and anti-tumor immunity to inhibit tumor progression in animal models of cancer.

Using *Itgam−/−* mice, as well as knockdown and neutralizing antibody approaches, we have demonstrated that integrin CD11b is not required for myeloid cell trafficking during tumor growth, although other studies have shown CD11b regulates myeloid cell recruitment under conditions of acute inflammation^17,18^. These differences may arise from the unique microenvironment cues and alterations in blood vessel biology observed in tumors versus inflamed tissue. Recent studies have shown that signaling pathways regulated by TLRs, CSF1R, PI3Kγ, and BTK control macrophage polarization^8–15^; here, we showed that CD11b promotes miRNA Let7a expression and inhibits Myc expression to control of macrophage polarization and tumor immune responses (Schematic, Fig. 5m). Taken together, these studies demonstrate that agonists of macrophage integrin CD11b could provide benefit in the treatment of cancer.

We observed improved vascular perfusion in CD11b−/− animals. Compared to non-pathological tissue, tumors display disorganized, and immature blood vessel structures. Tumor blood vessels often consist only of a single fenestrated endothelial layer and lack the additional coverage of mesenchymal cells, such as pericytes and smooth muscle cells, which provide blood vessels with a stable and more mature structure that promotes tumor perfusion and better access to chemotherapy^23–36^. In our studies, tumor blood vessels from CD11b−/− mice displayed increased pericyte coverage and increased vascular flow compared to WT. Indeed, deletion of CD11b or *Let7a* suppression increases the PDGF-BB/VEGF-A ratio, resulting in tumors with normalized vessels that stimulate tumor growth but are susceptible to cancer chemotherapy.

Integrin CD11b−/− macrophages expressed increased levels of IL-6 that induced the expression of STAT3-dependent immunosuppressive cytokines. Decreased integrin CD11b expression or activation negatively regulated the expression of miRNA *Let-7a* in macrophages, thereby upregulating intracellular *Il6* levels in macrophages. Reduced *Let-7a* expression is associated with malignant transformation of cancer cells and poor prognosis in cancer^40,41^. We found that loss of CD11b down-regulates *Let7a*, leading to elevated *Il6* levels and increased activation of STAT3, which were critical for the expression of M2-related cytokines. In contrast, recent studies showed that miRNA Let 7d-5p and DICER promote the M2 phenotype^42^. Together, these studies demonstrate the critical roles that miRNA species play in the polarization of macrophages and cancer growth.

Our studies show loss of Let7 expression in CD11b−/− macrophages induces Myc expression; Myc then drives immune suppressive transcription and inhibition of immune stimulatory transcription. Although both Myc and NFκB are transiently activated in WT IFNγ/LPS stimulated macrophages, NFκB activation is inhibited and Myc is activated in IFNγ/LPS stimulated *Itgam-/-* macrophages, leading to blockade of immune stimulatory transcription. These results suggest that Myc may inactivate NFκB in macrophages, thereby contributing to immune suppression and enhanced tumor growth

We found that LA1, a potent activator of integrin CD11b/CD18 in vitro and in vivo^42^, could significantly alter macrophage polarization, increase CD8+ T cell recruitment to tumors, inhibit tumor progression and prolong survival in mouse models of cancer. To determine the role of macrophage CD11b activation in tumor growth, we performed adoptive transfer of LA1 treated macrophages; LA1 treated macrophages directly and robustly inhibited tumor growth. LA1 effects were lost in *Itgam−/−* mice, indicating that the effects of LA1 on tumor growth depend on intact CD11b. These results indicate that LA1 acts on myeloid cells to effect changes in macrophage polarization, vasculogenesis and T cell recruitment. As LA1 has also been shown to inhibit bone marrow derived cell trafficking to tissues by promoting stable adhesion to endothelium. It is possible that LA1 may inhibit immune suppressive myeloid cell trafficking to tumors^49^. By slowing tumor progression, LA1 may also be useful to suppress the spread of cancer through metastasis.

We show that LA1 stimulates a pro-inflammatory phenotype in macrophages in vitro and in vitro. To address the direct impact of LA1 treated TAMs on tumor growth, we performed adoptive transfer of LA1 treated TAMs with tumor cells. We found that LA1 treatment of TAMs robustly and rapidly abolished tumor growth. In this model, we previously showed that adoptive transfer of immune suppressive macrophages (IL-4 stimulated) or TAMs promotes tumor growth, while adoptive transfer of immune stimulatory macrophages (IFNγ/LPS stimulated) and repolarized TAMs) recruited CD8+ T cells to tumors and rapidly abolished tumor growth^10^. We further showed that showed this tumor suppression by adoptively transferred macrophages required IL-12, a pro-inflammatory factor that recruits and stimulates CD8+ T cell proliferation^10^. Thus, we conclude by this evidence that LA1 repolarizes TAMs, leading to recruitment and activation of CD8+ T cells. As systemic delivery of LA1 also recruits CD8+ T cells and repolarizes TAMs in tumor models, these studies indicate that CD11b agonism by LA1 repolarizes macrophages and stimulates an adaptive immune response.

We tested the role of T cell depletion in the response to LA1 treatment by implanting MDA-MB-231 breast tumor cells in SCID mice, which lack T cells. By comparison, we implanted CL66 Luc breast tumor cells in syngeneic, immune competent mice. Interestingly, LA1 inhibited MDA-MB-231 tumor growth to 50% of that of control treated animals. In contrast, LA1 inhibited CL66 Luc tumor growth to 25% of that of control tumors. LA1 also repolarized macrophages by stimulating MHCII expression and reducing CD206, Arg1, MMP9 and S100A8 expression in TAMs and promoted CD8+ T cell recruitment in this model. Together, these results indicate that LA1 acts on myeloid cells to changes tumor associated macrophage polarization leading to increased T cell recruitment, and T cell dependent tumor suppression.

However, the partial effect of LA1 on MDA-MB-231 tumors indicates that LA1 (and hence CD11b) also affects T cell independent processes to suppress tumor growth. We showed that LA1 reduced inhibiting vascular normalization (as detected by pericyte coated blood vessel density) in all tumor models. Our studies indicate that CD11b controls innate immune cell polarization and that these cells regulate vascular as well as adaptive immune responses in the tumor microenvironment. Together, these results indicate that CD11 activation controls tumor macrophage polarization, vasculogenesis and T cell recruitment.

## Methods

### Materials

All materials used in this manuscript are publicly available

### Regulatory approvals

All animal experiments were performed with approval from the Institutional Animal Care and Use Committees of the University of California, San Diego, La Jolla, CA, Rush University Medical Center and the University of Miami Leonard M. Miller School of Medicine. All use of human peripheral blood leukocytes isolated from outdated leukophoresis samples from consented donors from the San Diego Blood Bank to make macrophages and use of human tumor cell lines were conducted with approval (NIH exempt category) from the Institutional Review Boards of the University of California, San Diego, La Jolla, CA, and Rush University Medical Center.

### Cell lines

C57BL/6 LLC, B16 melanoma, CL66 breast and MDA-MB-231 breast tumor cells were obtained from the American Type Culture Collection (ATCC). Cells were cultured in antibiotic- and fungizide-free DMEM or RPMI media containing 10% serum and tested negative for mycoplasma using the Mycoplasma Plus PCR primer set from Stratagene (La Jolla, CA). All cell lines were authenticated by tumor cell and tumor histology and by RNA or DNA sequencing.

### Mice

C57BL/6 and CD11b deficient (*Itgam−/−*) mice in the C57BL/6 background were from Jackson Laboratories. For the spontaneous breast cancer model, male PyMT+ mice on a C57BL/6 background were crossed with wild type (WT) or *Itgam−/*− C57BL/6 at the University of California, San Diego to generate WT and *Itgam*−/− P+ females. Wild type and *CD11b−/−* female mice heterozygous for the PyMT transgene were compared to each other. All PyMT+ females exhibit adenomas/early carcinomas by 16 weeks of age and late carcinomas by 25 weeks of age.

Generation of the ITGAM I332G knock-in mouse was accomplished by replacing exon 9 of the ITGAM gene using a targeting construct in which the Ile332 codon was substituted with Gly using a site-directed mutagenesis kit. The mutation led to the loss of the Exon-9 Bgl II restriction site. C57BL/6 ES cells with the heterozygous ITGAM I332G mutant allele were generated. G418-resistant clones were characterized by PCR, sequencing and southern blot analysis. The heterozygous mutant mice were generated using the blastocyst injection method. Mice heterozygous for the ITGAM I332G mutation were normal, fertile and phenotypically indistinguishable from wild-type (WT) littermates. The heterozygous mice were bred with ROSA26::FLPe knockin (JAX Stock No: 003946) mice to remove the selection cassette. The N2 offspring were backcrossed with C57BL/6N for six generations. The C57BL/6N N6 mice were crossed to obtain mice homozygous for the ITGAM I332G mutation that were also indistinguishable from wild-type (WT) littermates. Female nude and Balb/cJ mice at 6–8 weeks of age were from Jackson Laboratories and housed under pathogen-free conditions in the animal facility at Rush University Medical Center.

### Tumor studies

Subcutaneous tumor studies: 5 × 10^5^ LLC or B16 cells were injected subcutaneously into syngeneic (C57BL/6) 6 to 8-week old WT or *Itgam*−/− mice (*n* = 18). Tumors were excised at 7, 14, or 21 days, cryopreserved in OCT, lysed for RNA purification or collagenase-digested for flow cytometric analysis of CD11b+, F4/80+, and Gr1+ expression. Tumor volumes were calculated using the equation *v* = (*l*^*2*^ x *w*)/2. CL66-luc cells (0.5 × 10^6^ cells) were suspended in 50 μL of basement membrane extract Matrigel (BD Pharmingen) in PBS (1:1) and inoculated orthotopically in the fourth mammary fat pad of female Balb/cJ mice. Tumor dimensions were measured every 2 days, and tumor volume was calculated using the equation: *v* = π/6 (length) × (width), where length is the longest diameter of the tumor and width is the shorter diameter. Mice were divided into four cohorts: vehicle control treated, LA1 only treated, Taxol only treated, LA1 and Taxol treated. LA1 (2 mg/kg) was dissolved in 2% DMSO and 1% Tween-20 in saline and Taxol (2.5 mg/kg) was dissolved in a 1:1:6 ratio of Cremophor EL: Ethanol: Saline. LA1 was administered by intraperitoneal injection (i.p) daily, while Taxol was administered every other day by i.p injection until end-point. LA1 + Taxol treated mice received LA1 i.p in the morning followed by Taxol i.p within 4 h. Tumor burden was evaluated every other day by caliper measurements. Prior to tissue collection, mice were anesthetized by ketamine/xylazine and the lungs were perfused with PBS followed by 10% formalin and harvested for fixation. Mammary tumor tissue was harvested and divided for fixation (10% formalin or OCT), snap frozen, or made into single-cell suspensions and analyzed by flow cytometric analysis. For survival studies, Balb/c mice bearing CL66-derived tumors were treated for 6 weeks after the first treatment and monitored thereafter. End point was considered when the tumor reached 2 mm in diameter (*n* = 10–15). Alternatively, 5 million MDA-MB 231 human breast cancer cells were injected orthotopically in the 4th mammary fat pad of 6-week old female nude mice. The tumors were allowed to establish for 35 days (at least 0.5 cm in diameter) and the tumor bearing mice were divided into three treatment groups including, vehicle, LA1 only (i.p; 2.0 mg/kg; daily) and Taxol only (i.p; 2.5 mg/kg every other day). Treatment was initiated on day 35 and continued until the end-point at day 58 post-tumor when all the mice were sacrifice and tumor tissue was harvested for histological analysis. Palpable tumors were measured using digital calipers 3 times weekly during the entire experiment to develop tumor growth curves. LA1 treatment of LLC: 7.5 × 10^5^ LLC cells were injected subcutaneously (s.c) into the right flank of syngeneic 6 to 8-week old WT or CD11b−/− mice. At 5–8 days post tumor inoculation, tumor-bearing mice were divided into the following treatment groups: Vehicle (6% DMSO, 1% Tween-20 in saline), LA1 (6 mg/kg dissolved in vehicle). Vehicle and LA1 was administered by intraperitoneal injection (i.p) daily. Tumor burden was evaluated 2–3 times a week by caliper measurements and tumor volumes were calculated using the equation (*l*^2^ x *w*)/2. Tumors were harvested at 3 weeks post-tumor inoculation, formalin fixed, cryopreserved in OCT, or collagenase-digested for flow cytometric analysis. PyMT studies: The growth of spontaneous mammary tumors in PyMT+(*n* = 10) and *Itgam*−/−;PyMT+ (*n* = 14) animals in the C57Bl6 background was evaluated over the course of 0–25 weeks. All PyMT+ females exhibited adenomas/ early carcinomas by 16 weeks of age and late carcinomas by 25 weeks of age. Total tumor burden at endpoint was determined by subtracting the total mammary gland mass in PyMT− animals from the total mammary gland mass in PyMT+ animals.

ITGAM I332G knock-in mouse tumor studies: 7.5 × 10^5^ LLC cells were injected subcutaneously (s.c) into the right flank of syngeneic 6 to 8-week old WT or CD11b KI mice. Palpable tumors were established at 5–8 days post-tumor inoculation. Tumor burden was evaluated 2–3 times a week by caliper measurements and tumor volumes were calculated using the equation *v* = (*l*^2^ × *w*)/2. Tumors and spleens were harvested at 4 weeks post-tumor inoculation, weighed, formalin fixed or cryopreserved in OCT for histological analysis.

### Immunohistochemistry

Tumor samples were collected and cryopreserved in O.C.T. Sections (5 μm) were fixed in 100% cold acetone, blocked with 8% normal goat serum for 2 h, and incubated with primary antibodies at 1–5 µg/ml for 2 h at room temperature. Sections were washed 3 times with PBS and incubated with fluorescent secondary antibodies. Primary antibodies were: F4/80 (BM8, eBioscience), CD4 (H129.19, BD Bioscience), CD8 (53–6.7, BD Bioscience), CD31 (MEC13.1, BD Bioscience), desmin (RB0914-P1, LabVision, Thermo Scientific), and anti-smooth muscle actin (1A4, Sigma-Aldrich). Slides were counterstained with 4′,6-diamidino-2-phenylindole (DAPI) to identify nuclei. Immunofluorescence images were collected on a Nikon microscope (Eclipse TE2000-U) and analyzed using Metamorph image capture and analysis software (Version 6.3r5, Molecular Devices). Pixels/field or cell number/field were quantified in five 100× fields from 5 biological replicates. For LA1 studies, immunohistochemical staining was quantified by counting marker positive cells in 3 different areas analyzed at 40x using a light microscope for each tumor tissue (*n* = 4–6). Tissues that were stained with fluorochrome conjugated secondary antibodies were counter stained with DAPI and analyzed using the Zeiss 700 LSM confocal microscope and Zen software (Carl Zeiss Group, Hartford, Connecticut).

### Quantification of murine peripheral blood cells

To quantify myeloid cells in murine peripheral blood, blood was collected from naïve or tumor-bearing mice by retro-orbital bleeding into heparin-coated Vacutainer tubes (BD Bioscience), incubated in red blood cell lysis buffer. Cells were washed twice in PBS and stained for flow cytometric sorting.

### Isolation of single cells from murine tumors

Tumors were isolated, minced in a petri dish on ice and then enzymatically dissociated in Hanks Balanced Salt Solution containing 0.5 mg/ml Collagenase IV (Sigma), 0.1 mg/ml Hyaluronidase V (Sigma) and 0.005 MU/ml DNAse I (Sigma) at 37 °C for 5–30 min. The duration of enzymatic treatment was optimized for greatest yield of live CD11b+ cells per tumor type. Cell suspensions were filtered through a 70 μm cell strainer. Red blood cells were solubilized with red cell lysis buffer (Pharm Lyse, BD Biosciences, San Jose, CA), and the resulting suspension was filtered through a cell strainer to produce a single cell suspension. Cells were washed one time with PBS prior to use in flow cytometry analysis or sorting.

### Flow cytometry staining and analysis

Single cell suspensions (10^6^ cells in 100 µl total volume) were incubated with Aqua Live Dead fixable stain (Life Technologies, Carlsbad, CA), FcR-blocking reagent (BD Biosciences, San Jose, CA) and fluorescently labeled antibodies and incubated at 4 °C for 1 h. Primary antibodies were: BV605-F4/80 (clone BM8, Biolegend #123133, 1.25 µg/ml), Alexa 700-CD45 (clone 30-F11, eBioscience #56–0451, x 0.6 µg/ml), CD11b-APC (clone M1/70, eBioscience #17–0012, 0.3 µg/ml), FITC-Gr1 (clone RB6–8C5, eBioscience #11–5931, 3 µg/ml), eF780-CD3 (clone 145–2C11, eBioscience #47–0031, 5 µg/ml), PE-Dazzle-CD4 (clone RM4–5, Biolegend #100565, 0.4 µg/ml), and BV605-CD8 (clone 53–6.7, Biolegend #100743, 1.75 µg/ml). Multicolor FACS Analysis was performed on a BD Canto RUO 11 Color Analyzer. All data analysis was performed using the flow cytometry analysis program FloJo (Treestar).

### Human macrophage differentiation and culture

Human leukocytes concentrated by from apheresis were obtained from the San Diego Blood Bank. Cells were diluted in phosphate buffered saline (PBS), 0.5% BSA, 2 mM EDTA, incubated in red cell lysis buffer (155 mM NH_4_Cl, 10 mM NaHCO_3_ and 0.1 mM EDTA) and centrifuged over Histopaque 1077 to purify mononuclear cells. Approximately 10^9^ cells were purified by gradient centrifugation from one apheresis sample. Purified mononuclear cells were cultured in RPMI+ 20% serum+ 50 ng/ml Human M-CSF (PeproTech). Non-adherent cells were removed after 2 h by washing, and adherent cells were cultured for 6 days to differentiate macrophages fully.

### Murine macrophage differentiation and culture

Bone marrow derived cells (BMDC) were aseptically harvested from 6 to 8 week-old female mice by flushing leg bones of euthanized mice with phosphate buffered saline (PBS), 0.5% BSA, 2 mM EDTA, incubating in red cell lysis buffer (155 mM NH_4_Cl, 10 mM NaHCO_3_ and 0.1 mM EDTA) and centrifuging over Histopaque 1083 to purify the mononuclear cells. Approximately 5 × 10^7^ BMDC were purified by gradient centrifugation from the femurs and tibias of a single mouse. Purified mononuclear cells were cultured in RPMI+ 20% serum+ 50 ng/ml M-CSF (PeproTech).

### Macrophage polarization

Bone marrow derived macrophages were polarized with either IFNγ (20 ng/ml, Peprotech) plus LPS (100 ng/ml, Sigma) or LPS alone for 24 h or IL-4 (20 ng/ml, Peprotech) for 24–48 h. In some cases, macrophages were incubated with inhibitors of Stat3 (STAT4 VIII) or cMyc (10058-F4, Selleck). Total RNA was harvested from macrophages using the RNeasy Mini Kit (Qiagen) according to the manufacturer’s instructions. Secreted protein was measured in culture supernatants by ELISA assays.

### Analysis of gene expression

Total RNA was isolated from cells using RNeasy Mini Kit (Qiagen). cDNA was prepared using 1 µg RNA with the qScript cDNA Synthesis Kit (Quanta Biosciences) or the SuperScript III First-Strand Synthesis Kit (Invitrogen). Sybr green-based qPCR was performed using primers to murine *Gapdh (Mm_Gapdh_1_SG)*, *Arg1 (Mm_Arg_1_SG QT00134288) Tgfb (Mm_Tgfb_1_SG Qiagen QT00145250), Il10 (Mm_Il10_1_SG Qiagen QT00106169), Il6 (Mm_Il6_1_SG Qiagen QT00098875), Nos2 (Mm_Nos2_1_SG Qiagen QT00100275), Il12b (Mm_Il12b_1_SG Qiagen QT00153643)Ifng (Mm_Ifng_1_SG Qiagen QT01038821), Il1b (Mm_Il1b_2_SG Qiagen QT01048355), Tnfa (Mm_Tnfa_1_SG Qiagen QT00104006), Vegfa (Mm_Vegfa_1_SG Qiagen QT00160769), Pdgfb (Mm_Pdgfb_1_SG Qiagen QT00266910) and human GAPDH (Hs_GAPDH_1_SG Qiagen QT00079247)*, *ARG1 (Hs_Arginase_1_SG Qiagen QT00068446, IL6 (Hs_IL6_1_SG Qiagen QT00083720), NOS2 (Hs_NOS2_1_SG Qiagen QT00068740), IL12B (Hs_IL12B_1_SG Qiagen QT00000364), and PDGFB* (Hs_PDGFB_1_SG Qiagen QT00001260) (Qiagen QuantiTect Primer Assay). mRNA levels were normalized to *Gapdh or GAPDH* (ΔCt = Ct gene of interest – Ct *Gapdh*) and reported as relative mRNA expression (ΔΔCt = 2^-(ΔCt sample – ΔCt control)) or fold change.

### Immunoblotting

IL-4 and LPS macrophage cultures were solubilized in RIPA buffer containing protease and phosphatase inhibitors. Thirty µg protein was electrophoresed on Biorad precast gradient gels and electroblotted onto PVDF membranes. Proteins were detected by incubation with 1:1000 dilutions of primary antibodies, washed and incubated with Goat anti-rabbit-HRP antibodies and detected after incubation with a chemiluminescent substrate. Primary antibodies directed against NFκBp65 (D14E12, #8242 Cell Signaling Technology, 1:1000), pSer536NFκBp65 (93H1, #3033 Cell Signaling Technology, 1:1000), cMyc (D3N8F, #13987 Cell Signaling Technology, 1:1000) or pSer62 cMyc (E1J4K, #13748 Cell Signaling Technology, 1:1000). Anti-actin (#A2103 Sigma-Aldrich, 1:1000). Uncropped scans of Western blots are included as a supplementary figure in the Supplementary Information.

### In vivo macrophage adoptive transfer experiments

F4/80+ cells were isolated from single cell suspensions of 700–800 mg LLC tumors from donor WT or *Itgam*−/− mice by FACS sorting. Primary bone marrow derived macrophages from WT or *Itgam*−/− mice were polarized and harvested into a single cell suspension. Alternatively, WT tumor derived macrophages were incubated with LA1 or saline. Purified macrophages were admixed 1:1 with LLC tumor cells and 5 × 10^5^ total cells were injected subcutaneously into syngeneic host WT or *Itgam−/−* mice. Tumors were excised and weights were determined 14 days after inoculation.

### Integrin CD11b ligation experiments

Differentiated bone marrow derived macrophages were cultured on 5 µg/ml VCAM-1 or ICAM-1 (R&D Systems) coated culture plates or maintained in suspension. To inhibit integrin CD11b ligation, macrophages were cultured on 5 µg/ml ICAM-1 in the presence of 25 µg/ml anti-CD11b antibody (M1/70, BD Bioscience) or in suspension culture on BSA coated culture plates. As a control, macrophages were cultured in the presence of IgG control antibody.

### siRNA mediated knockdown

Differentiated macrophages were transfected (AMAXA, Mouse Macrophage Nucleofection Kit) using 100 nM of siRNA against *Itgam* (Mm_Itgam_01 or Mm_Itgam-05) or Il6 (Mm_Il6_01 or Mm_Il6_03) or non-silencing siRNA (Ctrl_AllStars_1) from Qiagen. After transfection, cells were cultured for 36–48 h in DMEM containing 10% serum and 10 ng/ml M-CSF (PeproTech). Efficiency of *Itgam* knockdown of each oligo was confirmed by quantitative RT-PCR QuantiTect Primer Assay) and flow cytometry.

### MicroRNA and anti-microRNA

Control miRNA, control anti-miRNA, Pre-miR-*Let7a* (PM10050) and Anti–miR-*Let7a* (AM10050) for in vitro studies were from Applied Biosystems. MicroRNA was delivered using siPORT (Ambion). To evaluate microRNA expression levels, total RNA was extracted with Trizol (Invitrogen), and RT-PCR was performed to detect let-7a (Mm_let-7a-1_2), let 7d (Mm_let-7d_1), or let7f (Mm_let7f-1_1) miScript Primer Assay. Data were normalized to the internal control small RNA *snoRNA202* (Applied Biosystems). For in vivo studies, oligomers were purchased from Sigma: Anti-miR scrambled control:5′[mG][mU][mC][mA][mA][mG][mG][mC][mA][mU]

[mC][mC][mG][mG][mA][mU][mC][mA][mU][mC][mA][mA]-3′

Anti-miR-Let7a:

5′[mA][mC][mU][mC][mC][mA][mU][mC][mA][mU][mC][mC][mA][mA][mC][mA][mU][mA][mU][mC][mA][mA]-3′

### Nanoparticle preparation and administration

The cyclic peptides, cRGDfK and cRADfK, were synthesized by using standard Fmoc solid-phase chemistry. Peptides were purified by reverse-phase HPLC, and mass was confirmed by mass spectroscopy. Peptides were conjugated to succinimidyl ester-(PEO)_4_-maleimide (Pierce). DSPE was reacted with iminothiolane (Sigma–Aldrich) to produce a free thiol. The DSPE containing the free thiol group was reacted with the cRGDfK-(PEO)_4_-maleimide or cRADfK-(PEO)_4_-maleimide to produce peptide-lipid conjugates.

Cholesterol/DOPE/DSPC/DSPE-(PEO)_4_-cRGDfK/DSPE-mPEG2000 (6:6:6:1:1 molar ratio) in chloroform was evaporated under argon gas and then hydrated in sterile 300 mM ammonium phosphate buffer (pH 7.4) at a total lipid concentration of 3.32 mM for 1 h. Liposomes were vortexed for 2–3 min and sonicated in ULTRAsonik 28× for 2–3 min at room temperature to produce multilamellar vesicles (MLVs). MLVs were then sonicated with a Ti-probe (Branson 450 sonifier) for 1–2 min to produce small unilamellar vesicles (SUVs). Stepwise extrusion was performed with the final step being extrusion through a polycarbonate filter with 100-nm pore size (Whatman). Liposomes incorporating cyclic peptides were used to form lipid-RNA complexes^43^. These complexes were formulated with a molar ratio of 4:1 calculated based on the *N*-[1-(2,3-dioleoyloxy)]-*N*,*N*,*N*-trimethylammonium propane (DOTAP) content of the liposomes. Nucleic acids (anti-miRNAs) and lipids were separately diluted in 100 μl RNase-free water. The RNA solution was added to the liposomes, mixed gently and the mixture was incubated at 25 °C for 5 min before injection into mice. Mice were treated with 5 µg of scrambled anti-miRNA or anti–miR-*Let7a* (Sigma) in RGD-nanoparticles intravenously every 3d starting from day 7 until the end of the experiment.

### Statistics

For studies evaluating mutations or drug treatments on tumor size, tumor volumes were computed and mice were randomly assigned to groups so that the mean volume +/− s.e.m. of each group was identical. A sample size of 10 mice/group provided 80% power to detect mean difference of 2.25 standard deviation (SD) between two groups (based on a two-sample *t*-test with 2-sided 5% significance level). Significance testing was performed by one-way Anova with Tukey’s posthoc testing for multiple pairwise testing or by parametric or nonparametric Student’s *t* test as appropriate. We used a two-sample *t*-test (two groups) and ANOVA (multiple groups) when data were normally distributed, a Wilcoxon rank sum test (two groups) when data were not and Fisher’s exact test when appropriate. All mouse studies were randomized and blinded; assignment of mice to treatment groups, tumor measurement and tumor analysis was performed by coding mice with randomly assigned mouse number, with the key unknown to operators until experiments were completed. In tumor studies for which tumor size was the outcome, animals removed from the study due to health concerns were not included in endpoint analyses.

## Electronic supplementary material

Supplementary Information

Description of Additional Supplementary Files

Supplementary Data 1

Reporting Summary

## Data Availability

All data generated or analyzed during this study available from the authors. The source data underlying all figures are provided in as a Source Data file (Supplementary Data 1). A reporting summary for this article is available as a Supplementary Information file.
