# Supplementary material for: Integrin CD11b activation drives anti-tumor innate immunity
Source: Nat Commun. 2018 Dec 19;9:5379. doi: 10.1038/s41467-018-07387-4 (PMC6300665; doi:10.1038/s41467-018-07387-4)
Supplement: Supplementary file 1 — Supplementary Information [file 41467_2018_7387_MOESM1_ESM.pdf]

Supplementary Figures

Schmid et al.

Integrin CD11b activation drives anti-tumor innate immunity

Supplementary Figure 1

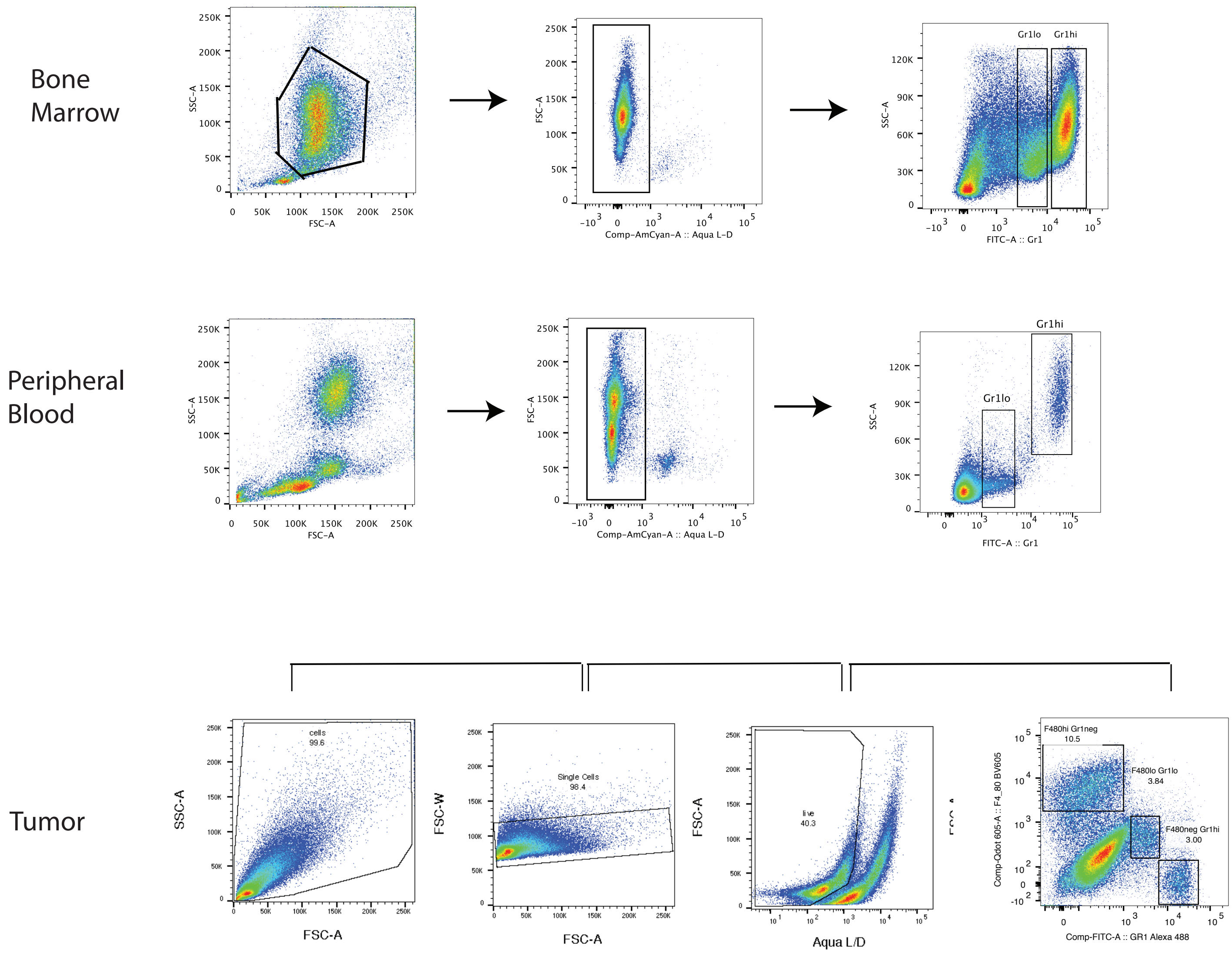

Supplementary Figure 1: Schematic of Immune profiles determined by flow cytometry

Flow cytometry analysis schematics for myeloid cells in bone marrow, peripheral blood and tumor from WT and *Itgam-/-* mice.

Supplementary Figure 2

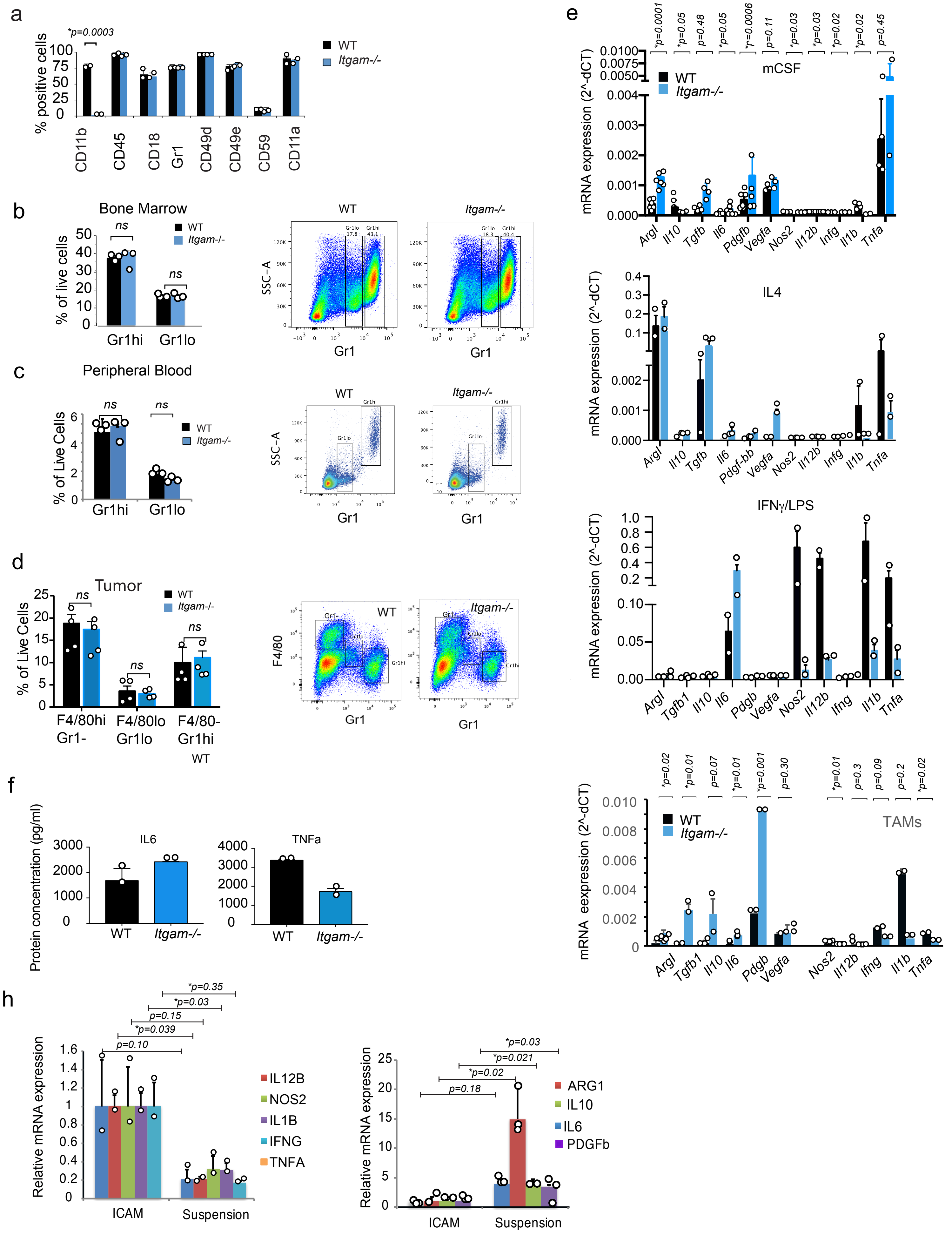

Supplementary Figure 2: Immune profiles of normal and tumor tissues of *Itgam-/-* mice

(**a**) Expression of immune cell markers and integrins in BM derived leukocytes from WT (black bars) and *Itgam-/-* (blue bars) mice (n=4). (**b-c**) Graphs and FACs profiles indicating proportions of $Gr1^{hi}$ granulocytes and $Gr1^{lo}$ monocytes in (**b**) bone marrow and (**c**) peripheral blood in WT (black bars) and *Itgam*-/- (blue bars) mice. (**c-d**) Graphs and FACs profiles indicating proportions of $Gr1^{neg}$ $F/480^{hi}$ macrophages, $Gr1^{lo}F/480^{hi}$ monocytes and $Gr1^{hi}F/480^{neg}$ granulocytes in LLC tumors from WT (black bars) and *Itgam*-/- (blue bars) mice. **(e)** mRNA expression of pro- and anti-inflammatory factors in basal, IL-4 and IFNγ/LPS-stimulated WT (black bars) and *Itgam*-/- (blue bars) in vitro cultured BM macrophages (n=4). **(f)** Concentrations (pg/ml) of IL-6 and TNFα secreted by WT (black bars) and *Itgam-/-* (blue bars) BMDM. (**g**) mRNA expression of pro- and anti-inflammatory factors in WT (black bars) and *Itgam*-/- (blue bars) TAMs from LLC tumors (n=3-4). (**h**) Relative mRNA expression of immune response genes in human macrophages adherent on ICAM-1 or maintained in suspension (n=3). Error bars indicate sem. "n" indicates biological replicates. *p (<0.05) indicates statistical significance by Student's t-test. Source data are provided as a Source Data file.

Supplementary Figure 3

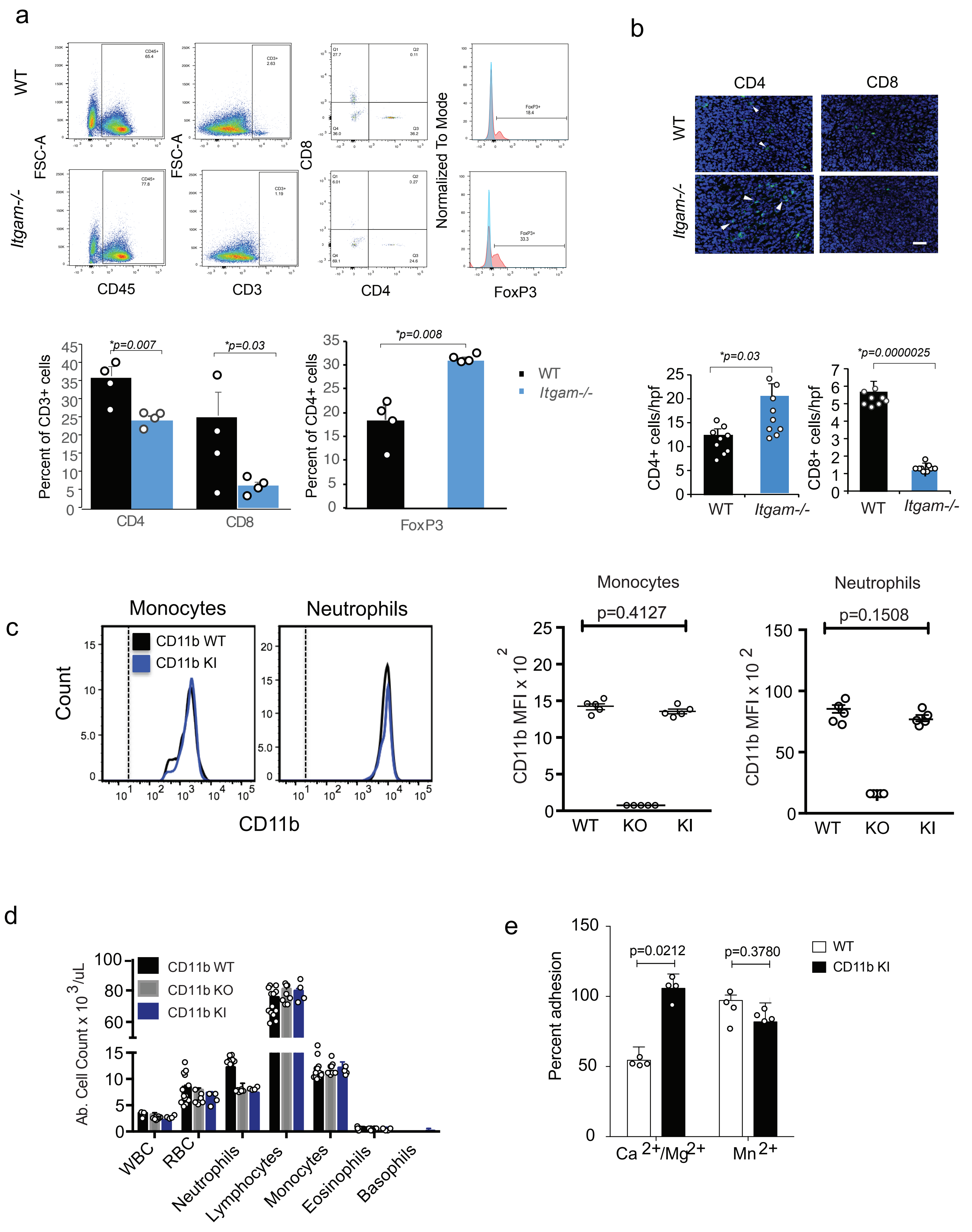

Supplementary Figure 3: Tumor derived cytokines suppress CD11b expression and promote immune suppressive macrophage phenotype

(**a**) Representative FACs profiles and quantification of CD4+, CD8+ and CD4+CD25+FoxP3+ T cells in WT (black bars) and *Itgam-/-* (blue bars) tumors (n=4). (b) Images and quantification of CD4 and CD8 immunostaining in LLC tumors from WT (black bars) and *Itgam*-/- (blue bars) mice (n=10). In images, bar indicates 50μm. (c) Representative FACs profiles of CD11b expression on neutrophils and monocytes from peripheral blood of *Itgam-/-* (KO) and *Itgam* I332G knockin (KI) mice; quantification of mean fluorescent intensities of CD11b on neutrophils and monocytes from peripheral blood of WT, *Itgam-/-* (KO) and *Itgam* I332G knockin (KI) mice (n=5). (d) Absolute numbers of white blood cells, red blood cells, neutrophils, lymphocytes, monocytes, eosinophils and basophils in WT (black bars, n=13) *Itgam-/-* (KO) (grey bars, n=8) and *Itgam* I332G knockin (KI) (blue bars, n=4) mice. (e) Percent adhesion to ICAM-coated surfaces of bone marrow derived macrophages from WT (white bars) and *Itgam* I332G knockin mice (black bars) in the presence of physiological concentration of Ca2+ and Mg2+ or in the presence of Mn2+ (n=4). Bar on micrographs indicates x 50 μm. n= number of biological replicates. *p (< 0.05) indicates statistical significance determined by Student's t-test (Supplementary Figure 3a-3b) and Mann-Whitney t-test (Supplementary Figure 3c-3e). Source data are provided as a Source Data file.

Supplementary Figure 4

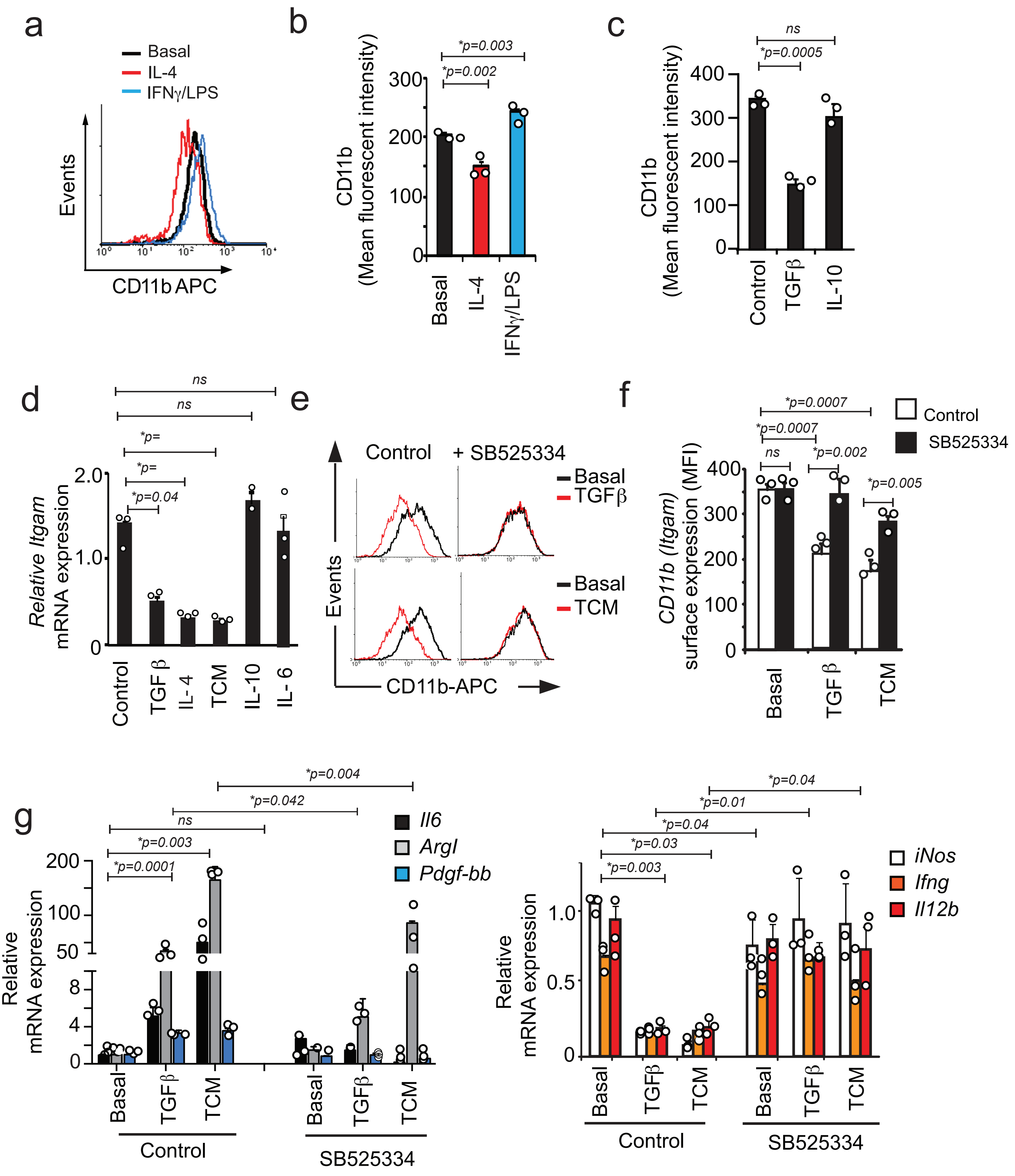

Supplementary Figure 4: Tumor derived cytokines suppress CD11b expression and promote immune suppressive macrophage phenotype

(**a**) FACS profile of CD11b expression levels in bone marrow derived macrophages stimulated with LPS/IFNγ (blue lines), IL-4 (red lines), or basal media (black lines) (n=3). (**b**) Quantification of CD11b mean fluorescence intensities (MFI) in bone marrow derived macrophages stimulated with LPS/IFNγ (blue bars), IL-4 (red bars), or basal (black bars) media (n=3). (**c**) Quantification of CD11b MFI in bone marrow derived macrophages stimulated with MCSF (Control), TGFβ, or IL10 (n=3). (**d**) *Cd11b* mRNA expression in WT bone marrow derived macrophages stimulated with TGFβ, IL-4, tumor conditioned medium (TCM), IL-10 or IL-6 (n=3). (**e**) FACS profiles of CD11b expression levels in bone marrow derived macrophages stimulated with basal media (black lines), TCM (red lines, lower panels) or TGFβ (red lines, lower panels) in the absence (Control, left panels) or presence (+SB525334, right panels) of the TGFβR1 inhibitor SB525334 (n=3). (**f**) Quantification of CD11b MFI in bone marrow derived macrophages stimulated with MCSF (basal) or TGFβ in the absence (control) or presence of the TGFβR1 inhibitor SB525334 (n=3). (**g**) Relative mRNA expression of WT and *Cd11b*-/- bone marrow derived macrophages stimulated with mCSF (basal), TGFβ or tumor conditioned medium (TCM) in the presence and absence of the TGFβR1 inhibitor (SB525334) (n=3). Error bars indicate sem. "n" indicates biological replicates. *p ($<$ 0.05) indicates statistical significance by Anova with Tukey's post-hoc testing (Supplementary Figure 4b-g). Source data are provided as a Source Data file.

Supplementary Figure 5

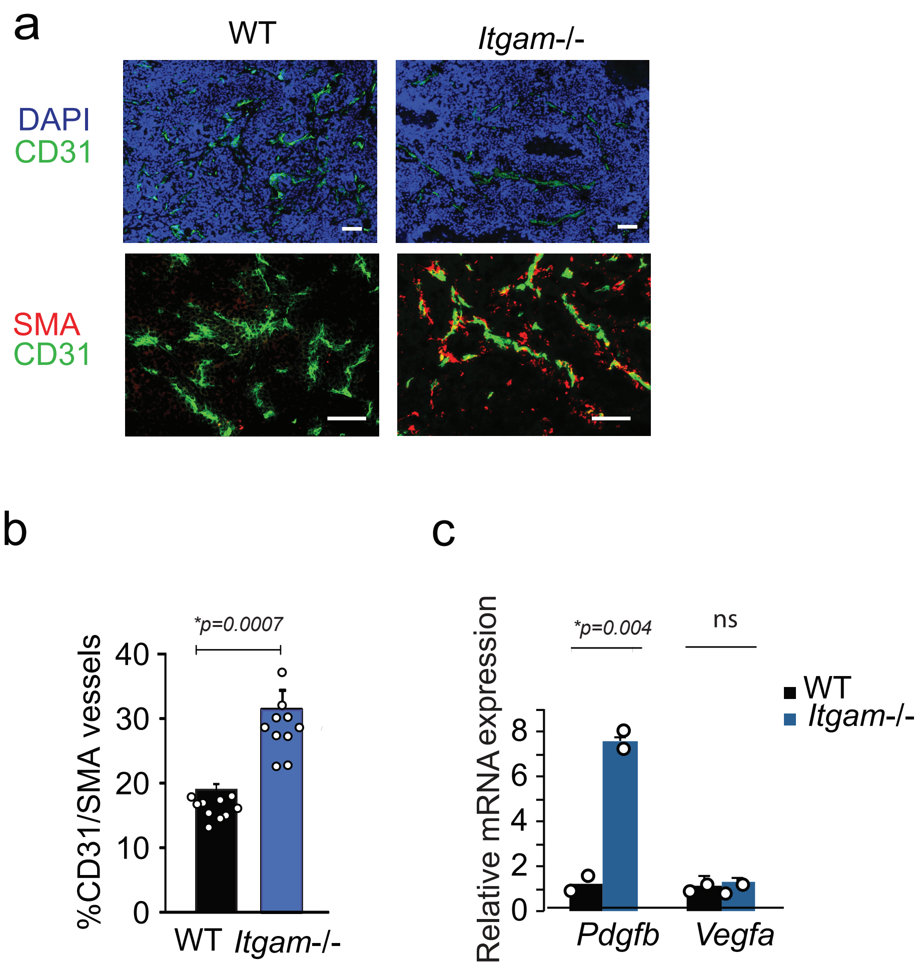

Supplementary Figure 5: CD11b loss promotes vascular normalization

(**a**) Images of CD31/DAPI and CD31/SMA immunofluorescence staining of mammary tumors from PyMT WT and *Itgam-/-* animals. Bar indicates 50 μm. (**b**) Percentage of CD31/SMA+ vessels in WT (black bars) and *Itgam-/-* (blue bars) tissues from a (n=10). (**c**) Relative mRNA expression of *Pdgfb* and *Vegfa* in mammary tumors from WT (black bars) and CD11b-/- (blue bars) *PyMT*+ animals (n=4). Error bars indicate sem. "n" indicates biological replicates. *p (<0.05) indicates statistical significance by Student's t-test (Supplementary Figure 5b-c). Source data are provided as a Source Data file.

Supplementary Figure 6

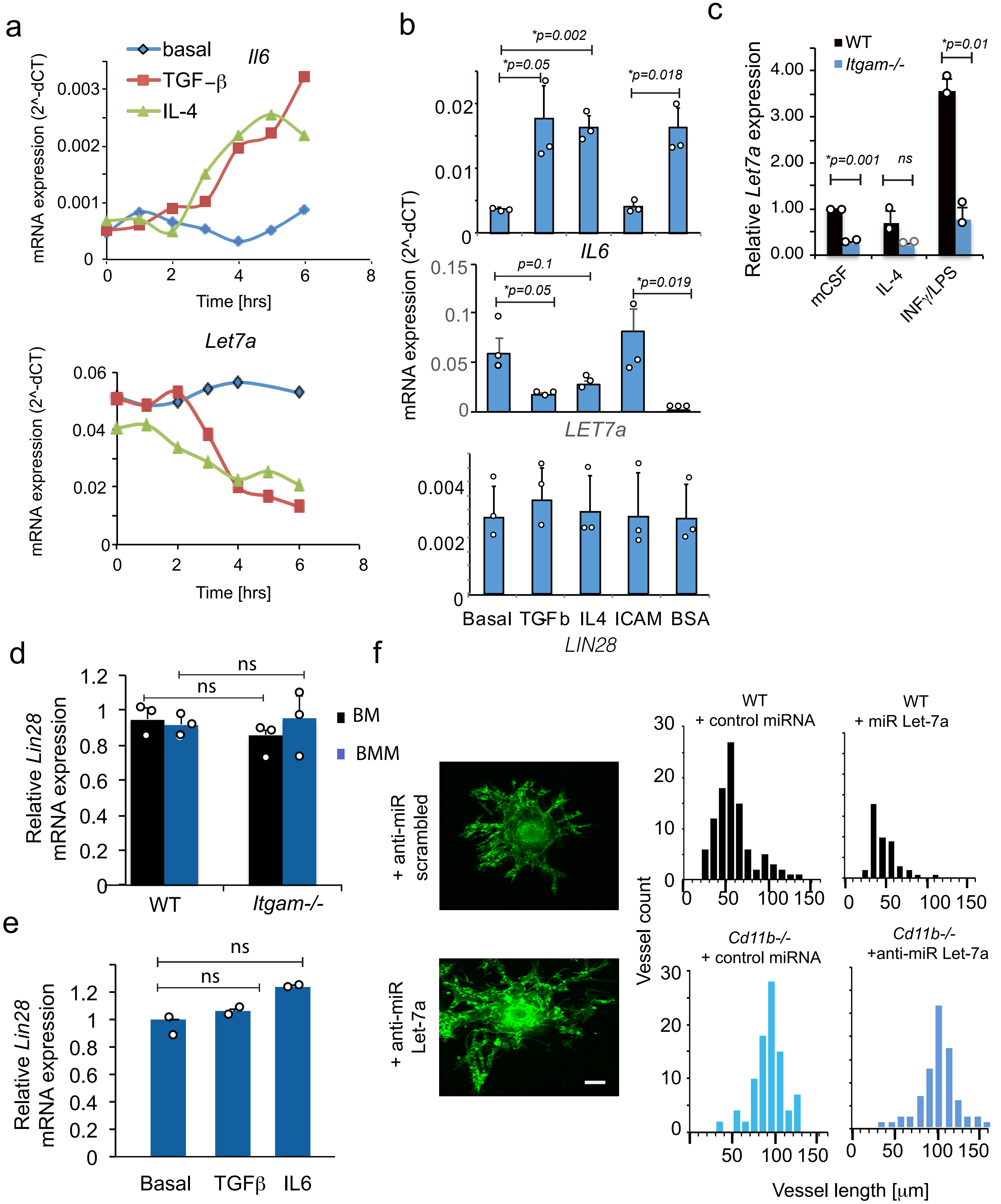

Supplementary Figure 6: Let 7a expression and role in macrophages

(**a**) Time course of expression of *Il6* mRNA (upper panel) or miRNA *Let7a* (lower panel) in mCSF-1 (basal, blue lines), TGFβ (red lines) or IL-4 (green lines) stimulated murine bone marrow derived macrophages in vitro (n=2). (**b**) Relative expression of *IL6, LET7a* and *LIN28* expression in human bone marrow derived macrophages treated with mCSF (basal), TGFβ or IL-4 or adherent to ICAM-1 vs in suspension (BSA) (n=3). (**c**) Relative expression levels of *Let7a* in mCSF-1 (basal), IL-4 or IFNγ/LPS stimulated WT (black bars) or *Itgam-/-* (blue bars) murine bone marrow derived macrophages in vitro (n=2). (**d)** Relative expression levels of Lin28 in total murine bone marrow myeloid cells (BM, black bars) or bone marrow derived murine macrophages (BMMs, blue bars) (n=3). **(e)** Relative expression levels of Lin28 in BMMs that were treated with basal medium, TGFβ or IL-6 (n=3). (**f**) Endothelial cells and vascular smooth muscle cells attached to microcarrier beads were cultured in fibrin gels containing WT or *Itgam*-/- BMMs transduced with control miRNA, miRNA *Let7a*, or anti-miRNA *Let7a*. Left: Images of representative anti-miR Let-7A and control-treated cultures. Right: Histograms of the lengths of CD31+ positive vessels in cultures containing WT (black bars) or *Itgam-/-* (blue bars) BMMs (n=10). Error bars indicate sem. "n" indicate biological replicates. *p (< 0.05) indicates statistical significance by Student's t-test for panels Supplementary Figure 6c, d, e. Anova with Tukey post-hoc testing for panel Supplementary Figure 6b. Source data are provided as a Source Data file.

Supplementary Figure 7

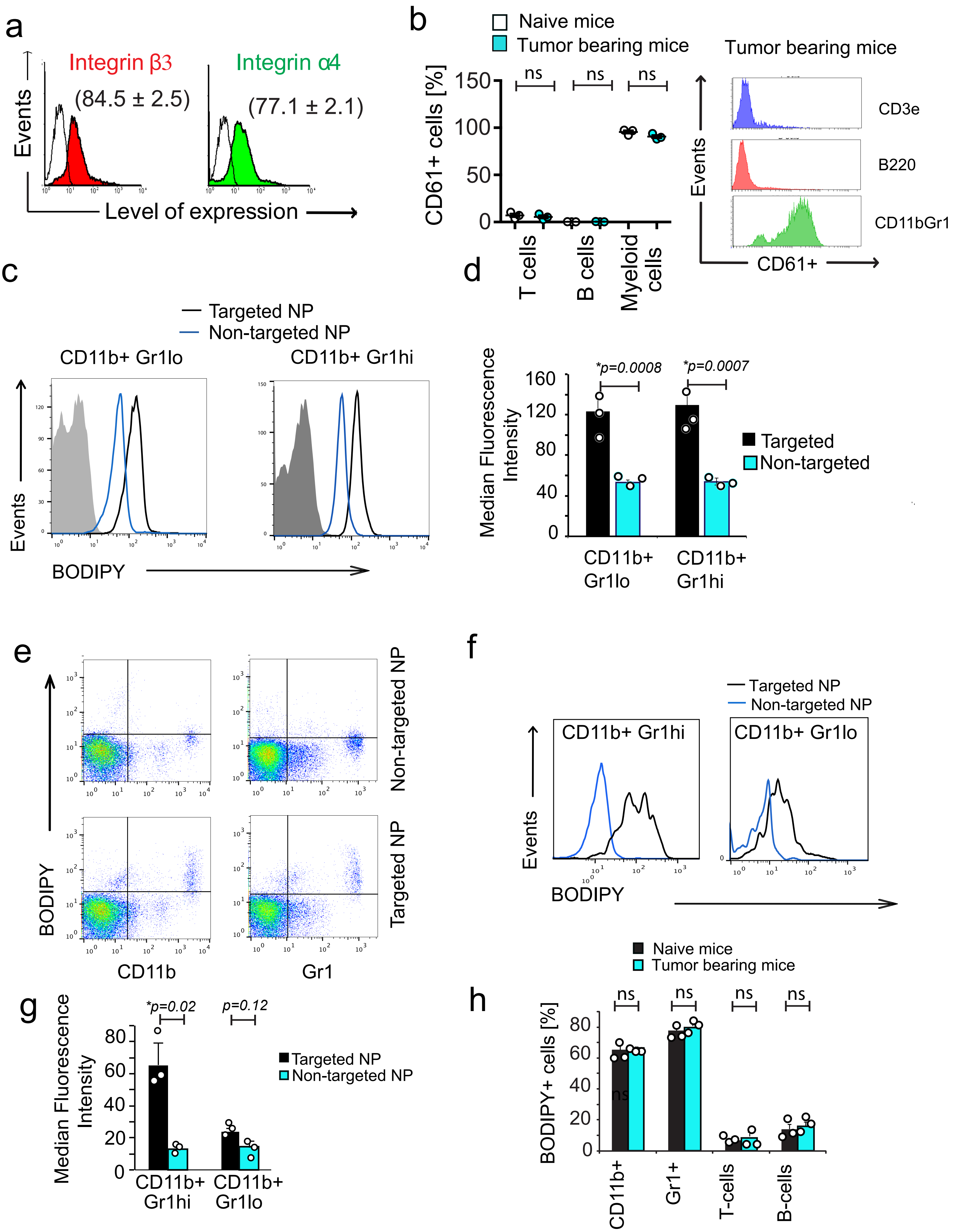

Supplementary Figure 7: RGD-coated nanoparticles target circulating and tumor associated myeloid cells.

(a) Representative flow cytometric profiles of control IgG (black line), anti-integrin α4 (green filled) and anti-integrin β3 (red filled) antibody immunostained murine peripheral blood CD11b+ myeloid cells. **(b)** Left, graph of percent integrin β3 (CD61) positive T, B and myeloid cells in circulation in naive (white bars) and LLC tumor bearing mice (cyan bars) (n=3). Right, representative histograms illustrating CD11b expression in these populations. **(c)** Representative BODIPY+ flow cytometric profiles of CD11b+Gr1lo monocytes and CD11b+Gr1hi granulocytes after in vitro administration of RGD-targeted (black lines) or non-targeted (blue lines) BODIPY-nanoparticles, compared to untreated cells (filled grey histogram). **(d)** Quantification of mean BODIPY+ fluorescence intensity induced in CD11b+Gr1lo monocytes and CD11b+Gr1hi granulocytes by incubation with RGD-targeted (black bars) or non-targeted (cyan bars) BODIPY-nanoparticles from **c** (n=3). **(e)** Representative flow cytometric profiles of BODIPY+ CD11b+ and Gr1+ peripheral blood mononuclear cells from tumor-bearing mice 2h after administration of RGD-targeted or non-targeted BODIPY-nanoparticles (n=3). **(f)** Histograms of relative levels of BODIPY uptake by RGD-targeted (black lines) or non-targeted (blue lines) BODIPY-nanoparticles in circulating CD11b+Gr1lo monocytes and CD11b+Gr1hi granulocytes from **e**. (**g**) Mean Fluorescence Intensity of RGD-targeted (black bars) or non-targeted (cyan bars) of BODIPY-nanoparticles in CD11b+Gr1lo monocytes and CD11b+Gr1hi granulocytes from **e** (n=3). (**h**) Quantification of percent BODIPY+ CD11b+myeloid cells, Gr1+myeloid cells, T cells and B cells in peripheral blood 2h after administration of RGD-targeted nanoparticles to naive (black bars) or tumor bearing

(cyan bars) animals (n=3). Error bars indicate sem. "n" indicates biological replicates. *p (< 0.05) indicates statistical significance by Student's t-test (Supplementary Figure 7d,7g,7h). Source data are provided as a Source Data file.

Supplementary Figure 8

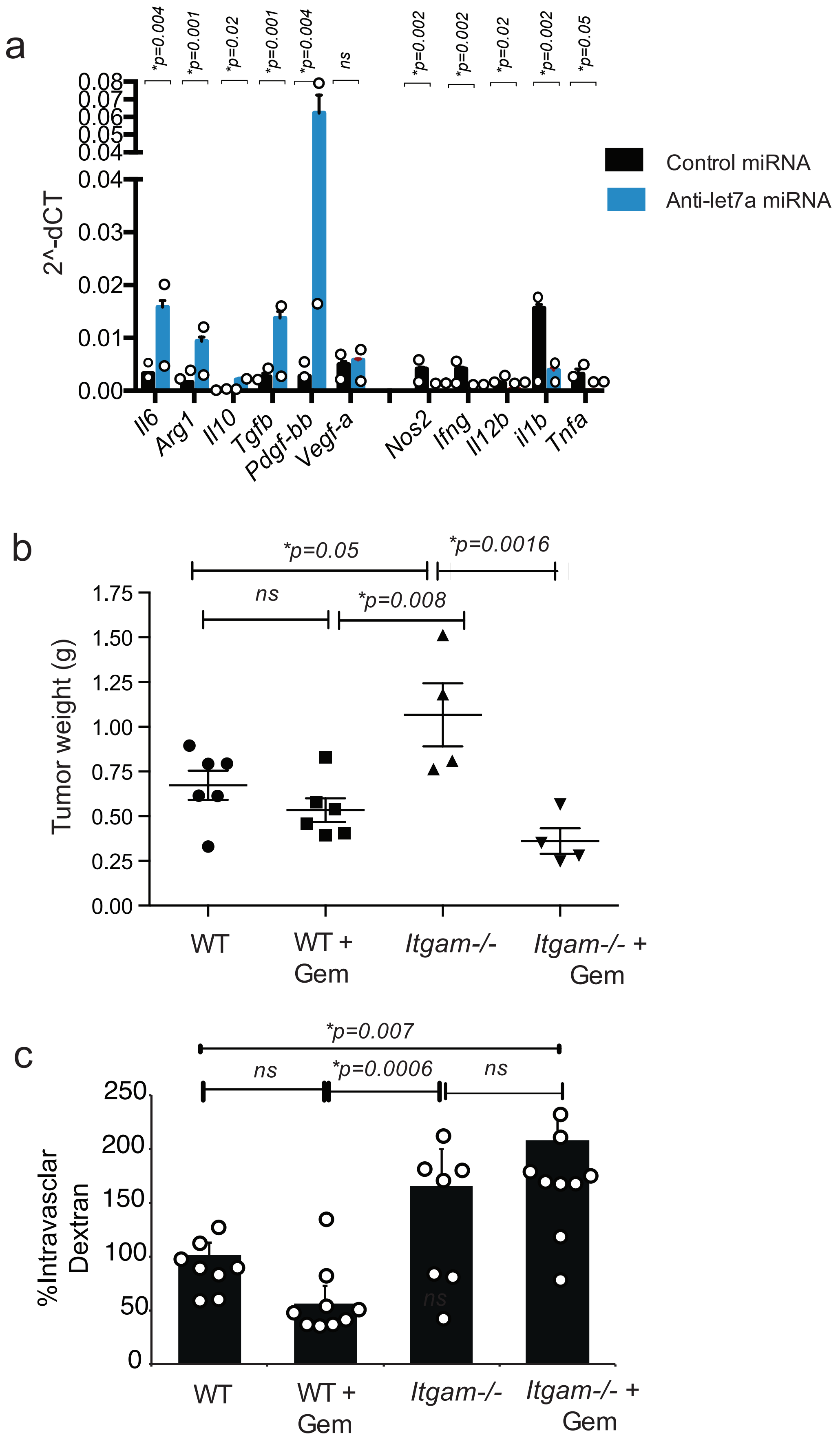

Supplementary Figure 8: Effect of Let7a and CD11b inhibition on tumors

(a) mRNA expression of inflammatory factors in sorted macrophages from LLC tumor-bearing mice treated with control miRNA (black bars) or anti-miR Let7a (blue bars)(n=3). (**b**) Weight of LLC tumors from WT and *Itgam-/-* mice treated with or without gemcitabine (n=4-6). (**c**) Percent perfusion (% intravascular FITC-dextran) in mice from (n=8 fields). **b**. Error bars indicate sem. "n" indicates biological replicates. *p (< 0.05) indicates statistical significance by Student's t-test for Supplementary Figure 8a and Anova with Tukey's post-hoc testing for Supplementary Figure 8b and 8**c**. Source data are provided as a Source Data file.

Supplementary Figure 9

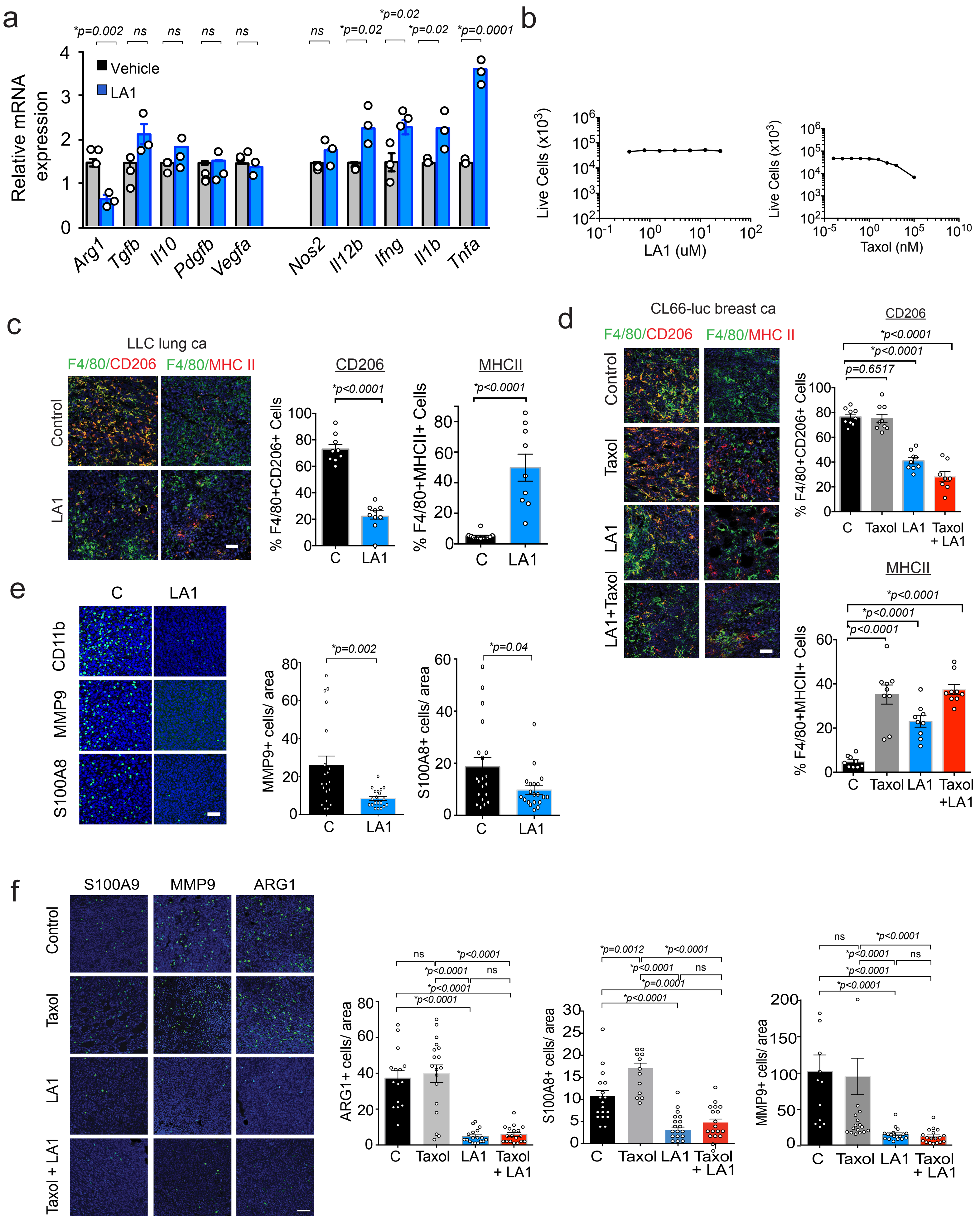

Supplementary Figure 9: Effects of LA1 on lung tumor progression

(**a**) mRNA expression of inflammatory cytokines in vehicle (grey bars) or LA1 (blue bars) treated IFNγ +LPS stimulated bone marrow derived murine macrophages (n=3). (**b**) Effect of LA1 (left) or taxol (right) on CL66-Luc breast tumor cell survival in vitro (n=3). (**c**) Images and quantification of CD206+ F4/80+ and MHC-II+ F4/80+ expression in LA1 (blue bars) and vehicle (black bars) -treated LLC tumors (n=5). (**d**) Images and quantification of CD206+F4/80+ and MHC-II+F4/80+ expression in vehicle (black bars), taxol (grey bars), LA1 (blue bars) and taxol + LA1 (red bars) -treated CL66-Luc tumors (n=5). (**e**) Images and quantification of CD11b, S100A9 and MMP9 expression in LA1 (blue bars) and vehicle (black bars) -treated LLC tumors (n=5). (**f**) Images of and quantification of LA1 (blue bars), taxol (grey bars), taxol + LA1 (red bars) and vehicle-treated (black bars) CL66-Luc tumor cryosections immunostained to detect S100A9, MMP9, and Arg1. Error bars indicate sem. "n" indicated biological replicates. *p (< 0.05) indicates statistical significance determined by Student's t-test for Supplementary Figure 9a, c, and e, and by Anova with Tukey's post-hoc testing for Supplementary Figure 9d and f. Source data are provided as a Source Data file.

Supplementary Figure 10

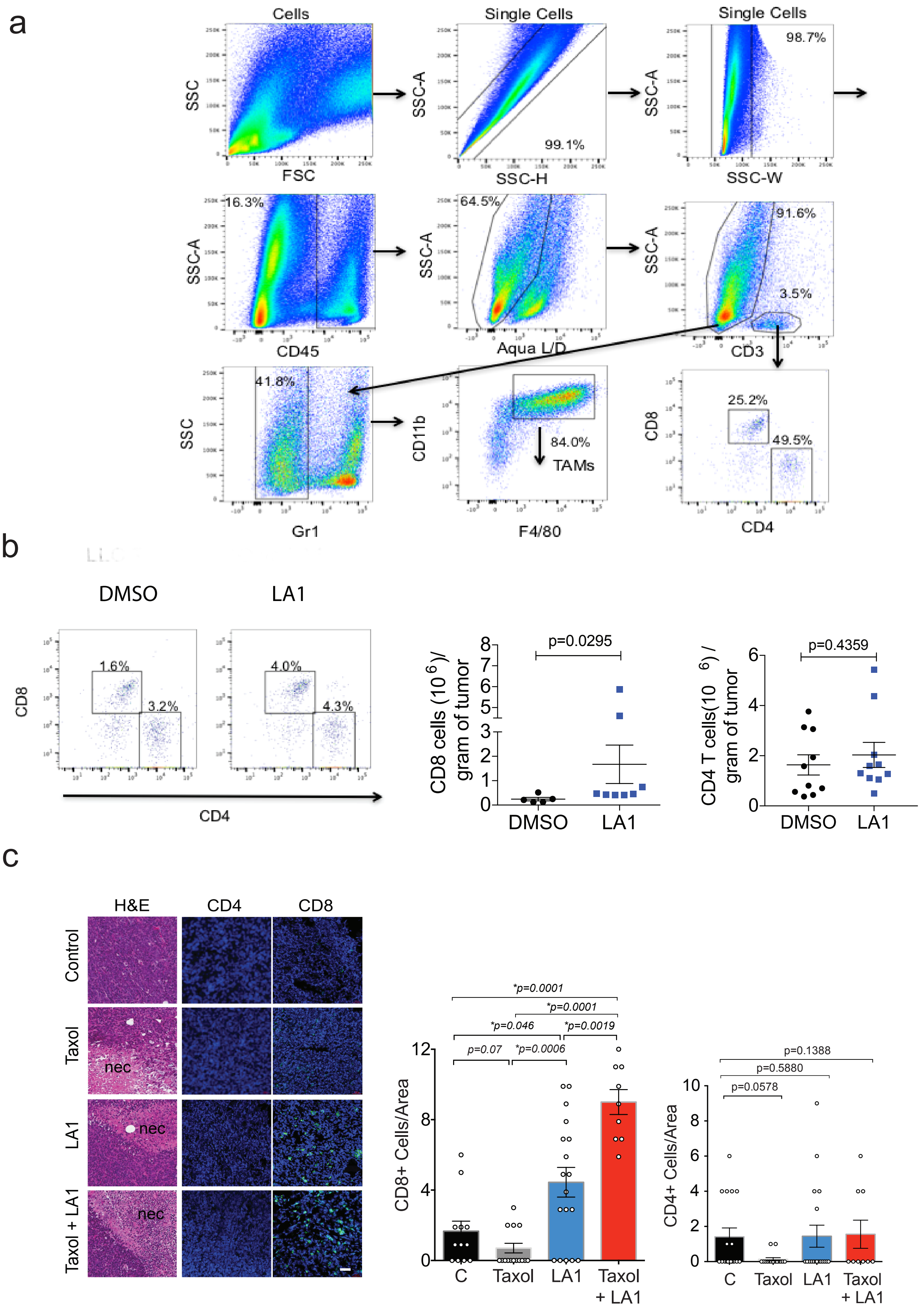

Supplementary Figure 10: Effects of LA1 on breast tumor progression

(**a**) Facs gating scheme for analysis of T cells in LA1 treated tumors. (**b**) Representative FACs profiles and quantification of CD4+ and CD8+ T cells in LA1 (blue dots, n=10) and vehicle (DMSO) treated (black dots, n=10) LLC tumors. (**b**) Images of and quantification of cryosections of CL66-Luc tumors from vehicle- (black bars), taxol (grey bars), LA1 (blue bars) and LA1 + taxol (red bars) treated tumors that were immunostained to detect CD4 and CD8 (n=8-16). Error bars indicate sem. "n" indicated biological replicates. Bar indicates 50 µm. *p (< 0.05) indicates statistical significance as determined by Student's t-test for Supplementary Figure 10b and by Anova with Tukey's post-hoc testing for Supplementary Figure 10c. Source data are provided as a Source Data file.
