# Supplementary material for: Integrin CD11b activation drives anti-tumor innate immunity
Source: Nat Commun. 2018 Dec 19;9:5379. doi: 10.1038/s41467-018-07387-4 (PMC6300665; doi:10.1038/s41467-018-07387-4)
Supplement: Supplementary file 2 — Description of Additional Supplementary Files [file 41467_2018_7387_MOESM2_ESM.docx]

**Description of Additional Supplementary Files**

**File Name**: Supplementary Data 1

**Description**: This file contains Source Data, comprised of one Excel workbook with one excel worksheets containing raw data corresponding to each figure panel. This file also contains original uncropped gel images found in figures.
