# Supplementary material for: Integrin CD11b activation drives anti-tumor innate immunity
Source: Nat Commun. 2018 Dec 19;9:5379. doi: 10.1038/s41467-018-07387-4 (PMC6300665; doi:10.1038/s41467-018-07387-4)
Supplement: Supplementary file 4 — Reporting Summary [file 41467_2018_7387_MOESM4_ESM.pdf]

nature research

Corresponding author(s): Varner, Judith A.

Reporting Summary

Nature Research wishes to improve the reproducibility of the work that we publish. This form provides structure for consistency and transparency in reporting. For further information on Nature Research policies, see Authors & Referees and the Editorial Policy Checklist.

Statistical parameters

When statistical analyses are reported, confirm that the following items are present in the relevant location (e.g. figure legend, table legend, main text, or Methods section).

n/a	Confirmed	
☐	☒	The exact sample size (*n*) for each experimental group/condition, given as a discrete number and unit of measurement
☐	☒	An indication of whether measurements were taken from distinct samples or whether the same sample was measured repeatedly
☐	☒	The statistical test(s) used AND whether they are one- or two-sided<br>*Only common tests should be described solely by name; describe more complex techniques in the Methods section.*
☐	☒	A description of all covariates tested
☐	☒	A description of any assumptions or corrections, such as tests of normality and adjustment for multiple comparisons
☐	☒	A full description of the statistics including central tendency (e.g. means) or other basic estimates (e.g. regression coefficient) AND variation (e.g. standard deviation) or associated estimates of uncertainty (e.g. confidence intervals)
☐	☒	For null hypothesis testing, the test statistic (e.g. *F*, *t*, *r*) with confidence intervals, effect sizes, degrees of freedom and *P* value noted<br>*Give P values as exact values whenever suitable.*
☒	☐	For Bayesian analysis, information on the choice of priors and Markov chain Monte Carlo settings
☒	☐	For hierarchical and complex designs, identification of the appropriate level for tests and full reporting of outcomes
☒	☐	Estimates of effect sizes (e.g. Cohen's *d*, Pearson's *r*), indicating how they were calculated
☐	☒	Clearly defined error bars<br>*State explicitly what error bars represent (e.g. SD, SE, CI)*

Our web collection on statistics for biologists may be useful.

Software and code

Policy information about availability of computer code

Data collection	n/a
Data analysis	n/a

For manuscripts utilizing custom algorithms or software that are central to the research but not yet described in published literature, software must be made available to editors/reviewers upon request. We strongly encourage code deposition in a community repository (e.g. GitHub). See the Nature Research guidelines for submitting code & software for further information.

Data

Policy information about availability of data

All manuscripts must include a data availability statement. This statement should provide the following information, where applicable:

- Accession codes, unique identifiers, or web links for publicly available datasets
- A list of figures that have associated raw data
- A description of any restrictions on data availability

Data Availability
All data generated or analyzed during this study available from the authors. The source data underlying all figures are provided in as a Source Data file. A reporting

summary for this article is available as a Supplementary Information file.

Field-specific reporting

Please select the best fit for your research. If you are not sure, read the appropriate sections before making your selection.

[x] Life sciences [ ] Behavioural & social sciences [ ] Ecological, evolutionary & environmental sciences

For a reference copy of the document with all sections, see nature.com/authors/policies/ReportingSummary-flat.pdf

Life sciences study design

All studies must disclose on these points even when the disclosure is negative.

Sample size	A sample size of 10 mice/group provided 80% power to detect mean difference of 2.25 standard deviation (SD) between two groups (based on a two-sample t-test with 2-sided 5% significance level). Significance testing was performed by one-way Anova with Tukey's posthoc testing for multiple pairwise testing or by parametric or nonparametric Student's t test as appropriate.
Data exclusions	No data were excluded.
Replication	Experiments were performed three or more times with multiple replicates per group.
Randomization	All mouse studies were randomized.
Blinding	Assignment of mice to treatment groups, tumor measurement and tumor analysis was performed by coding mice with randomly assigned mouse number, with the key unknown to operators until experiments were completed.

Reporting for specific materials, systems and methods

Materials & experimental systems

n/a	Involved in the study	
[ ]	[x]	Unique biological materials
[ ]	[x]	Antibodies
[ ]	[x]	Eukaryotic cell lines
[x]	[ ]	Palaeontology
[ ]	[x]	Animals and other organisms
[x]	[ ]	Human research participants

Methods

n/a	Involved in the study	
[ ]	[ ]	ChIP-seq
[ ]	[x]	Flow cytometry
[ ]	[ ]	MRI-based neuroimaging

Unique biological materials

Policy information about availability of materials

Obtaining unique materials	Itgam-/-; PyMT mice are available upon request.

Antibodies

Antibodies used	Primary antibodies were: : F4/80 (BM8, eBioscience), CD4 (H129.19, BD Bioscience), CD8 (53-6.7, BD Bioscience), CD31 (MEC13.1, BD Bioscience), desmin (RB0914-P1, LabVision, Thermo Scientific), and SMA anti-smooth muscle actin (1A4, Sigma-Aldrich), BV605-F4/80 (clone BM8, Biolegend #123133, 1.25 μg/ml), Alexa 700-CD45 (clone 30-F11, eBioscience #56-0451, x 10.6 μg/ml), CD11b (D11b-APC (clone M1/70, eBioscience #17-0012, 0.3μg/ml), FITC-Gr1 (clone RB6-8C5, eBioscience #11-5931, 3μg/ml), eF780-CD3 (clone 145-2C11, eBioscience #47-0031, 5μg/ml), PE-Dazzle-CD4 (clone RM4-5, Biolegend #100565, 0.4μg/ml), and BV605-CD8 (clone 53-6.7, Biolegend #100743, 1.75μg/ml) were from eBioscience, (D14E12,) #8242 Cell Signaling Technology, 1:1000), pSer536NF[illegible]Bp65 (93H1,) #3033 Cell Signaling Technology, 1:1000), cMyc (D3N8F, #13987 Cell Signaling Technology, 1:1000) or pSer62 cMyc (E1J4K,) or#13748 Cell Signaling Technology, 1:1000) were from Cell Signaling Technology. Anti-actin (#A2103 Sigma-Aldrich, 1:1000).
Validation	All antibodies were validated by their specific staining patterns in tissues by Western blots, IHC or FACs and compared to know patterns of staining in the literature.

Eukaryotic cell lines

Policy information about cell lines

Cell line source(s)	C57BL/6 LLC, B16 melanoma, CL66 breast and MDA-MB-231 breast tumor cells were obtained from the American Type Culture Collection (ATCC).
Authentication	All cell lines were authenticated by tumor cell and tumor histology and by RNA or DNA sequencing.
Mycoplasma contamination	All cell lines were routinely tested for mycoplasma contamination.
Commonly misidentified lines (See ICLAC register)	No cells lines were among the commonly misidentified lines.

Animals and other organisms

Policy information about studies involving animals; ARRIVE guidelines recommended for reporting animal research

Laboratory animals	laboratory mouse; C57Bl6; female and male; 6-15 weeks
Wild animals	*Provide details on animals observed in or captured in the field; report species, sex and age where possible. Describe how animals were caught and transported and what happened to captive animals after the study (if killed, explain why and describe method; if released, say where and when) OR state that the study did not involve wild animals.*
Field-collected samples	*For laboratory work with field-collected samples, describe all relevant parameters such as housing, maintenance, temperature, photoperiod and end-of-experiment protocol OR state that the study did not involve samples collected from the field.*

ChIP-seq

Data deposition

☐ Confirm that both raw and final processed data have been deposited in a public database such as GEO.

☐ Confirm that you have deposited or provided access to graph files (e.g. BED files) for the called peaks.

Data access links *May remain private before publication.*	*For "Initial submission" or "Revised version" documents, provide reviewer access links. For your "Final submission" document, provide a link to the deposited data.*
Files in database submission	*Provide a list of all files available in the database submission.*
Genome browser session (e.g. UCSC)	*Provide a link to an anonymized genome browser session for "Initial submission" and "Revised version" documents only, to enable peer review. Write "no longer applicable" for "Final submission" documents.*

Methodology

Replicates	*Describe the experimental replicates, specifying number, type and replicate agreement.*
Sequencing depth	*Describe the sequencing depth for each experiment, providing the total number of reads, uniquely mapped reads, length of reads and whether they were paired- or single-end.*
Antibodies	*Describe the antibodies used for the ChIP-seq experiments; as applicable, provide supplier name, catalog number, clone name, and lot number.*
Peak calling parameters	*Specify the command line program and parameters used for read mapping and peak calling, including the ChIP, control and index files used.*
Data quality	*Describe the methods used to ensure data quality in full detail, including how many peaks are at FDR 5% and above 5-fold enrichment.*
Software	*Describe the software used to collect and analyze the ChIP-seq data. For custom code that has been deposited into a community repository, provide accession details.*

Flow Cytometry

Plots

Confirm that:

☒ The axis labels state the marker and fluorochrome used (e.g. CD4-FITC).

☒ The axis scales are clearly visible. Include numbers along axes only for bottom left plot of group (a 'group' is an analysis of identical markers).

☒ All plots are contour plots with outliers or pseudocolor plots.

☒ A numerical value for number of cells or percentage (with statistics) is provided.

Methodology

Sample preparation	Tumors were isolated, minced in a petri dish on ice and then enzymatically dissociated in Hanks Balanced Salt Solution containing 0.5 mg/ml Collagenase IV (Sigma), 0.1 mg/ml Hyaluronidase V (Sigma),) and 0.005 MU/ml DNAse I (Sigma) or 1 mg/mL Collagenase A (Sigma) and 0.25 mg/ml DNAse I (Sigma)at 37°C for 5-30 min.
Instrument	Multicolor FACS analysis was performed on a BD Canto RUO 11 Color Analyzer or BD Fortessa.
Software	All data analysis was performed using the flow cytometry analysis program FloJo (Treestar)
Cell population abundance	Cell population abundance was verified by further facs analysis of a sample of sorted cells
Gating strategy	Gating strategy is described in the powerpoint.

☒ Tick this box to confirm that a figure exemplifying the gating strategy is provided in the Supplementary Information.

Magnetic resonance imaging

Experimental design

Design type	*Indicate task or resting state; event-related or block design.*
Design specifications	*Specify the number of blocks, trials or experimental units per session and/or subject, and specify the length of each trial or block (if trials are blocked) and interval between trials.*
Behavioral performance measures	*State number and/or type of variables recorded (e.g. correct button press, response time) and what statistics were used to establish that the subjects were performing the task as expected (e.g. mean, range, and/or standard deviation across subjects).*

Acquisition

Imaging type(s)	*Specify: functional, structural, diffusion, perfusion.*
Field strength	*Specify in Tesla*
Sequence & imaging parameters	*Specify the pulse sequence type (gradient echo, spin echo, etc.), imaging type (EPI, spiral, etc.), field of view, matrix size, slice thickness, orientation and TE/TR/flip angle.*
Area of acquisition	*State whether a whole brain scan was used OR define the area of acquisition, describing how the region was determined.*

Diffusion MRI ☐ Used ☐ Not used

Preprocessing

Preprocessing software	*Provide detail on software version and revision number and on specific parameters (model/functions, brain extraction, segmentation, smoothing kernel size, etc.).*
Normalization	*If data were normalized/standardized, describe the approach(es): specify linear or non-linear and define image types used for transformation OR indicate that data were not normalized and explain rationale for lack of normalization.*
Normalization template	*Describe the template used for normalization/transformation, specifying subject space or group standardized space (e.g. original Talairach, MNI305, ICBM152) OR indicate that the data were not normalized.*
Noise and artifact removal	*Describe your procedure(s) for artifact and structured noise removal, specifying motion parameters, tissue signals and physiological signals (heart rate, respiration).*
Volume censoring	*Define your software and/or method and criteria for volume censoring, and state the extent of such censoring.*

Statistical modeling & inference

Model type and settings

Specify type (mass univariate, multivariate, RSA, predictive, etc.) and describe essential details of the model at the first and second levels (e.g. fixed, random or mixed effects; drift or auto-correlation).

Effect(s) tested

Define precise effect in terms of the task or stimulus conditions instead of psychological concepts and indicate whether ANOVA or factorial designs were used.

Specify type of analysis: ☐ Whole brain ☐ ROI-based ☐ Both

Statistic type for inference
(See Eklund et al. 2016)

Specify voxel-wise or cluster-wise and report all relevant parameters for cluster-wise methods.

Correction

Describe the type of correction and how it is obtained for multiple comparisons (e.g. FWE, FDR, permutation or Monte Carlo).

Models & analysis

n/a	Involved in the study
☐	☐ Functional and/or effective connectivity
☐	☐ Graph analysis
☐	☐ Multivariate modeling or predictive analysis

Functional and/or effective connectivity

Report the measures of dependence used and the model details (e.g. Pearson correlation, partial correlation, mutual information).

Graph analysis

Report the dependent variable and connectivity measure, specifying weighted graph or binarized graph, subject- or group-level, and the global and/or node summaries used (e.g. clustering coefficient, efficiency, etc.).

Multivariate modeling and predictive analysis

Specify independent variables, features extraction and dimension reduction, model, training and evaluation metrics.
